# Acetylenic Substituent: Influence on the Structure, Electrochemical, Photophysical, and Thermal Properties of Rhenium(I) and Platinum(II) Complexes

**DOI:** 10.3390/molecules30040915

**Published:** 2025-02-16

**Authors:** Bartosz Zowiślok, Anna Świtlicka, Anna Maria Maroń, Sławomir Kula

**Affiliations:** Institute of Chemistry, University of Silesia, 9 Szkolna Str., 40-006 Katowice, Poland; bartosz.zowislok@us.edu.pl (B.Z.); slawomir.kula@us.edu.pl (S.K.)

**Keywords:** tricarbonyl rhenium(I) complex, platinum(II) complex, triimine ligand, photoluminescence, X-ray studies, electrochemistry, DFT calculations

## Abstract

The ‘wire-like’ acetylenic moiety is an important building block in organic and coordination chemistry that can facilitate electron transfer in donor–acceptor compounds, contributing to the enhancement of their photophysical properties. 2,6-Bis-(thiazol-2-yl)pyridine (*dtpy*) functionalized with a 4-phenylacetylene group (Ph-C≡C-*dtpy*) was, for the first time, used for the preparation of [ReCl(CO)_3_(Ph-C≡C-*dtpy*)] and [Pt(Ph-C≡C-*dtpy*)Cl]CF_3_SO_3_ in order to understand the properties derived from the use of the acetylenic substituent. The coordination ability of Ph-C≡C-*dtpy* toward Pt(II) and Re(I) centers was determined. All the studied compounds were characterized using FT-IR, ^1^H NMR, and ^13^C NMR spectroscopies, elemental analysis and, in the case of the free ligand and rhenium(I) complex, single-crystal X-ray analysis was also used. Their electrochemical, photophysical, and thermal properties were compared with the previously described similar systems. The photoluminescence spectra of Ph-C≡C-*dtpy*, [ReCl(CO)_3_(Ph-C≡C-*dtpy*)] and [Pt(Ph-C≡C-*dtpy*)Cl]CF_3_SO_3_ were investigated in solution and in the solid state at 298 K and 77 K. The experimental results were supported by the DFT and TD-DFT calculations. As a result of the introduction of the -C≡C- moiety into the organic ligand skeleton, the Re(I) and Pt(II) complexes display room-temperature emission. In the case of [Pt(Ph-C≡C-*dtpy*)Cl]CF_3_SO_3_, photoluminescence lifetime in a microsecond regime was observed, whereas nanosecond lifetime for [ReCl(CO)_3_(Ph-C≡C-*dtpy*)] in solution is comparable to lifetimes previously observed for rhenium(I) compounds with 4-substituted *dtpys*.

## 1. Introduction

The chemistry of transition metal complexes has attracted considerable attention in recent decades due to their numerous applications in various fields, including non-linear optical materials (NLO), solar energy conversion processes, chemical and biological sensors, catalysis, molecular electronics, supramolecular chemistry, and chemotherapy [1,2]. The luminescence properties of polypyridyl tricarbonyl rhenium(I) as well as platinum(II) complexes are among the scientific topics that have been explored for many years, because their photophysics is very complex. The Re(I) systems incorporating 2,2′:6′,2″-terpyridines (*terpys*) and its derivatives like 2,6-bis-(thiazol-2-yl)pyridines (*dtpys*) or 2,6-bis-(pyrazol-2-yl)pyridines (*dppys*) exhibit photophysical properties connected with various excited states, predominantly the metal-to-ligand-charge-transfer (MLCT) excited state, ligand-to-ligand-charge-transfer (LLCT) excited state, and intraligand (IL) excited state [3,4,5,6,7,8,9]. Research in recent years has shown that electron-rich groups introduced into the ligand skeleton lead to substantial changes in its photobehavior. The introduction of push–pull ligands into a metal environment allows for the emergence of efficient intraligand charge-transfer (ILCT) transitions from the donor orbital localized on the electron-rich group to the triimine-based π* acceptor orbital [10,11,12,13]. The proven approach to enhancing photophysical properties in solution is the functionalization of the 4-position in the *terpy* skeleton. In the case of both rhenium(I) and platinum(II) *terpy* compounds, the influence of electron-donating or electron-withdrawing substituents has been studied very extensively. However, complexes with *dtpys*, analogous to *terpy*-based systems, are much less known, but the impact of ligand substituents is also noticeable [5,6,7].

The photophysics of the ‘wire-like’ properties of the –C≡C– moiety, able to promote electron transfer in compounds as well as provide synthetic compatibility with polypyridyl-based ligand scaffolds, is well-known [14,15]. Interestingly, the impact of the triple bond on photophysical properties was also studied, not only for *terpy* derivatives, but also on substituted 2,2′-bipyridine (*bpy*) and 1,10-phenanthroline (*phen*). The approach, based on the extension of delocalization using an acetylenic substituent in order to favor highly allowed excited states with enhanced radiative transitions, was proven to be successful. Additionally, a series of soluble bis-*terpy* ligands were also found to be highly fluorescent [14]. Despite favorable prospects for improving optical properties, as well as synthetic compatibility with polypyridyl-based ligand scaffolds, to the best of our knowledge, there are only a few articles showing the luminescence properties of Ru(II) or Pt(II) derivatives [14,15,16,17,18]. Castellano et al. postulated that, in the family of Pt(II) compounds, the presence of acetylenic linkage in the 4-position of the *terpy* skeleton should lower the energy of the MLCT excited state and promote the luminescence of Pt(II) complexes in solution. Another benefit was the greatly extended excited state lifetimes, because low-lying triplet states of conjugated ligand structures can configurationally mix with charge-transfer triplets, resulting in a variety of possible triplet states. As expected, Pt(II) terpyridyl acetylene chloride complexes show long lived photoluminescent excited states at room temperature (τ; 0.010–0.016 µs) [19]. On the other hand, in disubstituted [Ru(4′-phenylethynyl-*terpy*)(CH_3_CN)_3_](PF_6_)_2_, the impact of the substituent on lifetime is rather negligible [17]. Low et al. studied series of ethynyl–phenylene-substituted *terpy* ligands (L), which were used to synthesize four complexes of general formula [RuCl(bpy)(L)]PF_6_ [15]. The electronic spectra, supported by TD-DFT calculations, confirmed that the ethynyl–phenylene moiety has an impact on the excited state in all Ru(II) compounds by tuning the composition of the LUMO, while the HOMO is rather metal-centered. Unfortunately, no photophysical studies were performed for this series of compounds.

To the best of our knowledge, photophysical studies focusing on transition metal complexes based on *dtpys* with acetylenic linkages remain unexplored. While *dtpys* and their complexes have received some attention, they are often overshadowed by the more extensively studied *terpy*-based systems [9,20,21,22,23,24,25]. Here, we report for the first time the synthesis, structural characterization, and electronic structure, as well as the electrochemical, photophysical, and thermal properties, of two complexes: [ReCl(CO)_3_(Ph-C≡C-*dtpy*)] and [Pt(Ph-C≡C-*dtpy*)Cl]CF_3_SO_3_, both based on 4-phenylacetylene-*dtpy* (Ph-C≡C-*dtpy*).

## 2. Results and Discussion

### 2.1. Synthesis and General Characterization

**Figure 1:**
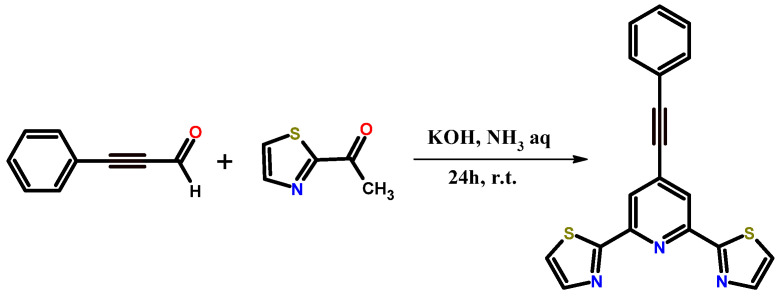
Synthesis of Ph-C≡C-*dtpy* by condensation.

4-phenylacetylene-2,6-bis(thiazol-2-yl)pyridine (Ph-C≡C-*dtpy*) was obtained by the condensation of phenylpropargyl aldehyde and 2-acetylthiazole in the presence of potassium hydroxide and ammonia (Figure 1). The synthesized ligand was purified by crystallization. The use of phenylpropargyl aldehyde allowed for the direct introduction of a triple bond into the structure of the final compound. Interestingly, the literature has not described the synthesis of *dtpy* derivatives containing an acetylene linker by condensation. In the synthesis of pyridine derivatives (especially *terpy* derivatives) containing a triple bond in their structures, Sonogashira coupling is most often used. However, despite high yields, this method requires a catalytic system and time-consuming purification methods [16,26,27,28,29]. Therefore, simple and cheap condensation is an exciting alternative to the coupling reaction mentioned above. (Figure 1)

The orange solid [ReCl(CO)_3_(Ph-C≡C-*dtpy*)] was synthesized by heating equimolar mixtures of the *dtpy* derivative and [Re(CO)_5_Cl] in toluene. The [Pt(Ph-C≡C-*dtpy*)Cl]CF_3_SO_3_ complex was synthesized according to a modification of the Büchner’s procedure [30,31,32,33,34]. In a two-step method, the precursor [PtCl_2_(PhCN)_2_] was refluxed in acetonitrile with one equivalent of AgCF_3_SO_3_ to yield the intermediate complex [PtCl(PhCN)_2_(CH_3_CN)]CF_3_SO_3_. After the filtering of AgCl, the intermediate complex was treated with Ph-C≡C-*dtpy* in a solvothermal reaction to obtain the desired compound. The FT-IR spectra of the free ligand show characteristic bands in the range of 1591–1492 cm^−1^, assigned to the ν(C=N) and ν(C=C) stretching vibrations. For metal complexes, these vibrations are only slightly shifted in comparison with those reported for Ph-C≡C-*dtpy*. Moreover, [ReCl(CO)_3_(Ph-C≡C-*dtpy*)] displays two overlapping lower-energy bands (1919 and 1890 cm^−1^) and a sharp, intense band at 2022 cm^–1^, attributed to the ν(C≡O) of the *fac*-[Re(CO)_3_]^+^ moiety. Absorptions assignable to the sulfonate part of the CF_3_SO_3_^–^ anion occur at 1262 cm^−1^ (ν_a_(SO_3_)), 1029 cm^−1^ (ν_s_(SO_3_)), and 636 cm^−1^ (δ_a_(SO_3_)) [35] (see Figure S1, Supplementary Materials). Consistent with the bidentate coordination mode of Ph-C≡C-*dtpy*, the resonances attributed to the outer thiazol-2-yl protons are clearly differentiated in ^1^H NMR spectra. Distinctive signals corresponding to the carbonyl groups appear in the range of 195.70 ppm, 193.59 ppm, and 188.98 ppm in ^13^C NMR spectra. Detailed ^1^H NMR analysis of 4-phenylacetylene-2,6-bis(thiazol-2-yl)pyridine (Ph-C≡C-*dtpy*) showed that thiazole rings are observed in the spectrum as two doublets with a characteristic coupling constant (in the range of 3.1–3.2) at values of 7.95 ppm and 7.49 ppm, respectively. The signal from the central pyridine ring can be observed as an intense singlet at a value of 8.29 ppm. The remaining signals in the ^1^H NMR spectrum originating from the substituent (specifically from the phenyl ring) are observed as two multiplets at values of 7.59–7.55 ppm and 7.42–7.38 ppm. In the case of the ^13^C NMR spectrum, we see a series of signals originating from carbon atoms located in individual aromatic rings. Particularly noteworthy are two signals at 95.31 ppm and 86.56 ppm, respectively, originating from the triple bond (Figure S2).

### 2.2. Molecular Structure

**Figure 2:**
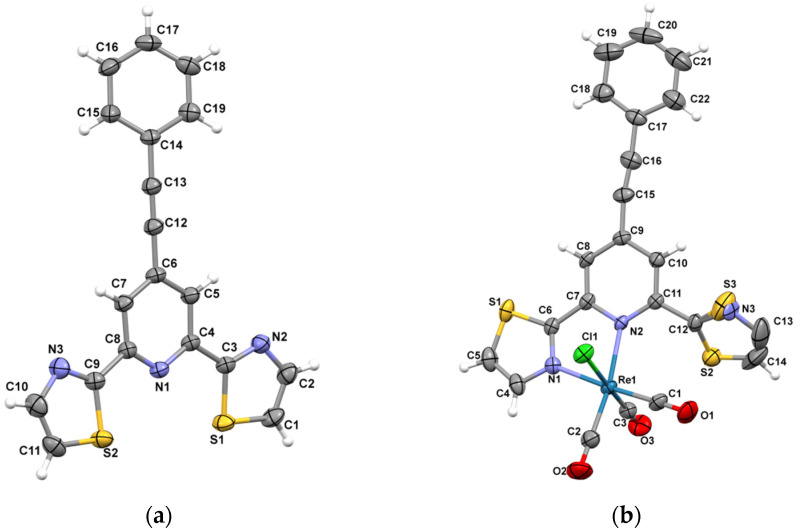
A perspective view showing the asymmetric units of Ph-C≡C-dtpy (**a**) and [ReCl(CO)_3_(Ph-C≡C-dtpy)] (**b**) with atom numbering. The displacement ellipsoids are drawn at 50% probability.

Ph-C≡C-*dtpy* and [ReCl(CO)_3_(Ph-C≡C-*dtpy*)] crystallize in the monoclinic P2_1_/*c* and P2_1_/*n* space groups, respectively. The perspective view of its molecular structure together with the atom numbering is shown in Figure 2, while crystal data together with the selected bond distances and angles are collected in Tables S1 and S2 in the Supplementary Materials.

**Figure 3:**
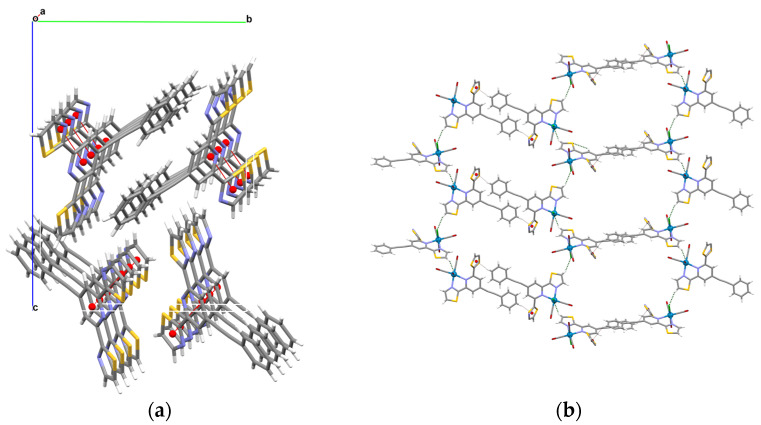
A view of the packing of Ph-C≡C-*dtpy* showing intermolecular π–π stacking interactions (**a**); a view of supramolecular honeycomb-like layers in [ReCl(CO)_3_(Ph-C≡C-*dtpy*)] (**b**).

The *dtpy* skeleton in Ph-C≡C-*dtpy* is almost planar, and the dihedral angles between the central pyridine plane and two peripheral ones are 4.68° and 7.05°. The substituent plane is inclined to the central pyridine ring at 22.02°, and such a small twist angle is the result of the triple –C(12)≡C(13)– bond system. The C–C, C–S, and C–N bond lengths (Table S2) in Ph-C≡C-*dtpy* lie within the expected ranges. The molecules of the free ligand are linked into supramolecular chains by π–π stacking involving the pyridyl and thiazolyl rings of the *dtpy* skeleton [3.8480(13) Å for Cg(1) S(2)/C(9)/N(3)/C(10)/C(11)⋯Cg(2) N(1)/C(4)/C(5)/C(6)/C(7)/C(8); (b) = −1 + *x*, *y*, *z*] (Figure 3a, Table S4).

The rhenium(I) cation in [ReCl(CO)_3_(Ph-C≡C-*dtpy*)] adopts a distorted octahedral coordination geometry, defined by three carbonyl ligands in a *fac* arrangement, one chloride atom, and two nitrogen atoms of chelating Ph-C≡C-*dtpy*. Due to the steric influence of the uncoordinated thiazole ring, the Re–N_py_ bond length to the central pyridine (Re(1)-N(2) = 2.236(4) Å) is longer than the corresponding Re–N_thiazol_ distance to the thiazolyl ring (Re(1)-N(1) = 2.167(4)Å). This structural dependence is observed in related rhenium(I) compounds [5,9,36,37].

Moreover, the bidentate coordination of Ph-C≡C-*dtpy* is responsible for the strong distortion of the coordination angles in [ReCl(CO)_3_(Ph-C≡C-*dtpy*)]. The presence of a five-membered chelate ring of *dtpy* derivative results in an N(1)–Re(1)–N(2) angle of 74.79(14)°, which is significantly smaller than the ideal 90° angle.

C(1)–Re(1)–N(2) equals 101.71(18)° and is the largest angle in comparison to the other *cis*-located angles. Finding the sum of the bond angles around the rhenium(I) center, which is 1620° for an ideal octahedral ligand arrangement, is the best method to show the degree of distortion of the local geometry; for the studied structure, this value is 1603.63° [38]. The crystal packing analysis (Mercury 2.4 program) demonstrates that the molecules [ReCl(CO)_3_(Ph-C≡C-*dtpy*)] are extended into supramolecular honeycomb-like layers through C(20)–H(20)∙∙∙π [X–H∙∙∙Cg = 3.607(11) Å] and C(5)–H(5)∙∙∙Cl(1) [D∙∙∙A distance 3.563(5)Å; D–H∙∙∙A angle = 150.00°] types of interactions. (Figure 3b, Tables S2 and S5 in Supplementary Materials) [39].

### 2.3. Electronic Absorption Spectra

The UV-Vis spectra of Ph-C≡C-*dtpy* were recorded in solution (2.5 ∙ 10^−5^ M) in three solvents exhibiting different dielectric constants: acetonitrile (ε = 37.5), chloroform (ε = 4.8), and DMSO (ε = 45.0).

**Figure 4:**
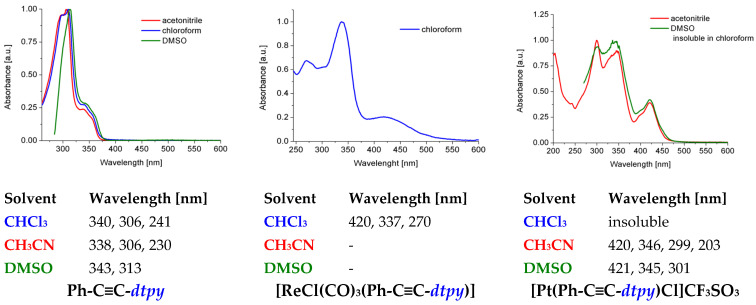
UV-VIS spectra together with experimental data.

The absorption spectra of the free ligand present a collection of bands in the range of 350–200 nm, assigned to the π–π* transition bands. The lowest energy bands are marginally affected by the change in solvent polarity, so it can be assumed that the ground and excited states have a similar dipole moment and their charge-transfer character is negligible. The absorption bands in the higher energy region of 346–196 nm can be attributed to the intraligand (IL) π→π* and n→π* transitions of the Ph-C≡C-*dtpy* ligand (Figure 4).

**Figure 5:**
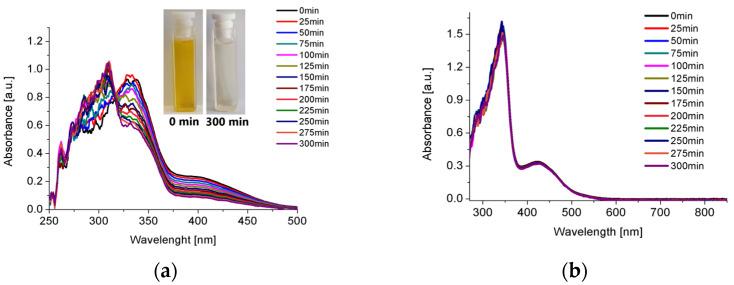
UV-Vis spectra of [ReCl(CO)_3_(Ph-C≡C-*dtpy*)] in acetonitrile (**a**) and chloroform (**b**) recorded once every 25 min for 5 h.

There was no change in the absorbance profile of the designed Pt(II) complex over 5 h, indicating its stability in solutions. A tricarbonylrhenium(I) complex in CH_3_CN and DMSO began to decompose after 25 min, but the compound is stable in chloroform (Figure 5 and Figure S4). The CHCl_3_ solution of Re(I) and the CH_3_CN solution of Pt(II) show a broad absorption band at 420 nm, which in both cases can be attributed to the MLCT character in both compounds (Figure 4).

**Figure 6:**
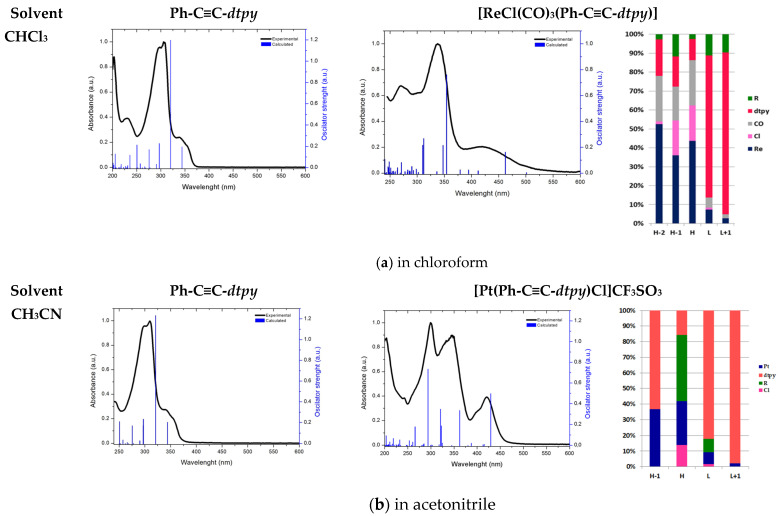
Experimental (blue line) absorption spectra in chloroform (**a**) and acetonitrile (**b**), along with transitions (black lines) computed at the TD-DFT/PBE1PBE/def2-TZVP/def2-TZVPD level, together with the compositions of selected frontier orbitals for metal complexes.

In order to get more insight into the nature of the electronic transitions involved in the absorption processes, computational studies at the TD-DFT/PBE1PBE/def2-TZVP/def2-TZVPD level were undertaken [40]. The calculations were carried out in two solvents: chloroform and acetonitrile. As shown in Table S2, the predicted bond lengths and angles of the free ligand and rhenium(I) complex are in good agreement with the values based upon the X-ray crystal structure data, and the general trends observed in the experimental data are well reproduced in the calculations, providing confidence regarding the reliability of the chosen method to reproduce the geometries of the studied complexes. The experimental and calculated electronic absorption spectra of the free ligand and both metal complexes are compared in Figure 6.

The lowest energy absorption band of the Re(I) and Pt(II) complexes is generally attributed to three allowed electronic transitions corresponding to one-electron excitation: HOMO→LUMO, H-1→LUMO, H-2→LUMO, HOMO→L + 1 for the Re(I) complex, and HOMO→LUMO, H-1→LUMO, and HOMO→L + 1 for the Pt(II) compound. The π* orbitals localized on the *dtpy* moiety dominated LUMO and LUMO + 1 in both complexes (74% and 85% in Re(I), and 82% and 97% in Pt(II)compounds). In the Re(I) complex, the HOMO, H-1, and H-2 are composed of π localized mainly on the metal center, with compositions of 44%, 36%, 52.5%, respectively. The percentage of ligands in all three orbitals does not exceed 25%. In the Pt(II) complex, significant participation of the π orbitals of the R substituent and *dtpy* in HOMO and HOMO-1 is shown by compositions of 58% and 63%, respectively. The percentage contributions of the metal(II) ion in these orbitals are 28% and 36%. In summary, in the first described compound, all the transitions can be assigned to MLCT (dπ_Re_→π*_Ph-C≡C-*dtpy*_), with a significant admixture of ILCT transitions (charge transfer with electronic density originating from CO groups, Cl atom, and R substituent unit to the π-conjugated *dtpy* acceptor moiety). However, in Pt(II)derivative, the situation is the opposite, and the transitions can be ascribed to dominant ILCT with little participation of MLCT (dπ_Pt_→π*_Ph-C≡C-*dtpy*_) (see Figures S3 and S5, Tables S6–S8).

### 2.4. Photoluminescence

The photoluminescence properties of the free ligand, [ReCl(CO)_3_(Ph-C≡C-*dtpy*)], and [Pt(Ph-C≡C-*dtpy*)Cl]CF_3_SO_3,_ were thoroughly investigated in CHCl_3_ and CH_3_CN solutions at room temperature, as well as in the solid state as powder and thin film and in a 4:1 ethanol-methanol (4:1 *v*/*v*) glass matrix at 77K. The photoluminescence data are summarized in Table 1 and Figure S6 in the Supplementary Materials.

Excitation of Ph-C≡C-*dtpy* in solution (CHCl_3_ and CH_3_CN) resulted in emission bands without vibronic structure, with a maximum at ~386 nm, which remained unaffected by changes in solvent polarity. The emission lifetimes of the free ligand fall within the nanosecond time regime, while luminescence quantum yields are in the range of 18.83–21.27%. The solid-state emission spectra of the free ligand at room temperature showed a structureless band with the maximum red-shifted with respect to the values reported for solutions. The intermolecular coupling present in the *dtpy* derivative seems to be responsible for shifting the emissions toward longer wavelengths. With reference to the solution, both luminescent lifetime and quantum yield are enhanced in the solid state.

Excitation of [ReCl(CO)_3_(Ph-C≡C-*dtpy*)] at the lowest absorption band gave rise to a broad emission band, with a maximum at 720 nm in CHCl_3_. Similar to other tricarbonyl rhenium compounds, the studied Re(I) complex also exhibits relatively weak emissive lifetimes in solution (Table 1). In the solid state and rigid glass at 77 K, the emission bands are broad and structureless, with the maxima blue-shifted in comparison to those in chloroform.

This can be attributed to the rigidochromic effect responsible for raising the energy of the emissive ^3^MLLCT due to the lack of solvent reorganization following excitation. Compared to the solution, the luminescent lifetimes and quantum yields are significantly increased. At 77 K, the Re(I) complex displays a microsecond excited-state lifetime, indicating that the emission originates from the excited triplet state. Decreasing the temperature leads to an increase in τₑₘ, consistent with the raising of the ^3^MLCT level, which causes a hypsochromic shift in emission and reduces the rates of non-radiative ^3^MLCT→S_0_ transitions [41,42,43]. The irradiation of [Pt(Ph-C≡C-*dtpy*)Cl]CF_3_SO_3_ gave rise to a structured emission band with maxima in the yellow-orange region (580, 620 nm). The acetonitrile solution of the Pt(II) complex at ambient conditions displayed rather weak emission bands, with a quantum yield of 1.91%.

The calculated transitions corresponding to excitation wavelengths are HOMO→LUMO, H-1→L, and H→L + 1, according to Table S7 in the Supplementary Materials. On this basis, it can be concluded that the excited state has an ILCT character, with a slight admixture of MLCT. The powdered sample [Pt(Ph-C≡C-*dtpy*)Cl]CF_3_SO_3_ displayed a relatively long-lived emission and enhanced quantum yield in comparison to its solution data. It is a well-known feature of platinum(II) complexes that the aggregation-induced phenomenon is usually responsible for the enhanced properties (Φ and τ) of emission in the solid state [44]. The solid-state emission spectrum of the sample at room temperature showed no vibronic band, with the maximum largely bathochromically shifted with respect to the value reported in acetonitrile (100 nm). In this case, the presence of Pt⋯Pt interactions and/or Pt–π stackings seems to be the reason for such a large shift [45].

In order to understand the optical properties of Ph-C≡C-*dtpy* and its compounds, we decided to compare them with *dtpy* derivatives found in the literature, including rhenium(I) and platinum(II) compounds, focusing on the effect of the R substituent at the 4-position, with varying electron-donating or electron-withdrawing properties. A brief overview of the substituents used to date in the free ligands on the *dtpy* backbone and their coordination compounds, together with the most important photophysical parameters, is shown in Tables S10–S12 in the Supplementary Materials. Generally, with a few exceptions, functionalized *dtpys* display rather longer wavelength emissions, and they are more emissive compared to Ph-C≡C-*dtpy*. Only in the case of phenyl– and naphtyl substituents is the emission wavelength very slightly blue-shifted by approximately 6 nm in comparison to Ph-C≡C-*dtpy*. As can be expected, the introduction of the conjugated 9-anthryl, 9-phenantryl, or 1-pyrenyl moieties into the *dtpy* skeleton significantly shifts the emission bathochromically. A comparison of the 4-phenyl-*dtpy*, known from the literature [Tables S10–S12], with the currently studied 4-phenylacetylene-*dtpy*, indicates that the changes in quantum yield and luminescence lifetime are relatively subtle. In the case of the phenyl substituent, the Φ values measured in acetonitrile and chloroform are slightly higher compared to Ph-C≡C-*dtpy* [24.77% (CH_3_CN) and 25.67% (CHCl_3_) for phenyl derivative; 18.83% (CH_3_CN); 21.27%(CHCl_3_)]. However, the lifetimes in Ph-C≡C-*dtpy* are slightly longer compared to the latter substituent. In the family of rhenium(I) tricarbonyl complexes of general formula [ReCl(CO)_3_(4-R-*dtpy*)], it can be seen that in chloroform solution the quantum yields, staying in the range of 0.28–1.3%, are lower compared to [ReCl(CO)_3_(Ph-C≡C-*dtpy*)], with Φ = 8.39%. In the solid state, no similar trend can be distinguished. In the case of platinum(II) compounds, the literature describes only three *dtpy*-based Pt(II) complexes, which at room temperature show a structureless phosphorescence band in dichloromethane; however, in polar solvents (DMSO and CH_3_CN), the phosphorescence is the subject of solvent-induced exciplex quenching.

### 2.5. Electrochemical Properties

The electrochemical properties of the studied compounds were investigated in DMF solution by cyclic voltammetry (CV) under argon atmosphere, using a glass carbon disk as the working electrode, a silver wire as the quasi-reference electrode, a platinum coil as an auxiliary electrode, and ferrocene as a standard, assuming that the IP of ferrocene equals −5.1 eV [46]. Cyclic voltammograms of the compounds are shown in Figure S7 in the Supplementary Materials. The spectroscopic and electrochemical data for the free ligand and coordination compounds are listed in Table 2. For all compounds, reversible reductions were observed, but the oxidation process is beyond the solvent limit and could not be determined. The voltammograms for all compounds display two reduction processes in the negative potential range, occurring at more positive values in comparison with the corresponding free ligand. The first reversible reduction is observed at −2.00 V for the ligand, and −1.38 V and −0.95 V are observed for the Re(I) and Pt(II) complexes, respectively. Compared to [Pt(*terpy*)Cl]CF_3_SO_3_ (−1.24 V) and [Pt(PhC≡C-*terpy*)Cl]CF_3_SO_3_ (−1.13 V), the first reduction peak of [Pt(Ph-C≡C-*dtpy*)Cl]CF_3_SO_3_ is anodically shifted, indicating that the Pt(II) complex with *dtpy* is easier to reduce [19,47].

With reference to the previous electrochemical studies for rhenium(I) tricarbonyls, they may be interpreted as ligand-based reductions. In most cases, the LUMO of these complexes are usually located in the *dtpy* derivative. Compared to other *dtpy*–Re(I) systems, the reduction of the studied complex is easier, because the first reduction potentials are less negative, which corresponds to lower LUMO levels. In the literature data of [ReCl(CO)_3_(4-R-*dtpy*)], R substituents show more negative reduction potentials (from −1.60 to 1.74 V), which can be attributed to the electron-donor effect induced by the substituent group. A similar situation can be observed in the case of Pt(II) complexes; however, electrochemical studies of the complexes with *dtpy* are rather rarely described (Table 3).

### 2.6. Thermal Properties

In order to examine the thermal properties of the studied compounds, thermogravimetric analysis (TGA) and differential scanning calorimetry (DSC) were applied, and the results are collected in Table S9 in the Supplementary Materials. As expected, the free ligand decomposed in a single step at T_max_ = 288 °C. Between the two 3D coordination compounds, the Pt(II) complex is more thermally stable compared to the rhenium(I) system. The decomposition temperatures T_5%_ (corresponding to 5% weight loss) and T_10%_ (10% weight loss) are commonly used as criteria for determining the thermal stability of compounds. In the case of [Pt(Ph-C≡C-*dtpy*)Cl]CF_3_SO_3_, the T_5%_ and T_10%_ values are approximately ~70 °C higher than those measured for the Re(I) complex (T_5%_ = 277 °C, T_10%_ = 325 °C). The thermal decomposition of the Pt(II) complex proceeds in two steps, in contrast to the multi-step decomposition observed in the second compounds. Comparing the examined rhenium(I) system to other compounds based on *dtpy* derivatives, it can be observed that both the Re(I) complex and free ligand are characterized by relatively low T_5%_and T_10%_ values. For [ReCl(CO)_3_(4-Ph-*dtpy*)] and [ReCl(CO)_3_(4-pyrph-*dtpy*)] (*4-pyrph* = 4-(4-pyrrolidinephenyl)), the T_5%_ and T_10%_ values fall within the range of 312–350 °C and 328–376 °C, respectively, while their organic ligands remain stable at temperatures ranging from 255 to 344 °C and 270–365 °C, respectively [6]. It can be concluded that the introduction of a 4-phenylacetylene substituent into the skeleton of *dtpy* results in a decrease in the thermal stability of [ReCl(CO)_3_(Ph-C≡C-*dtpy*)] and Ph-C≡C-*dtpy*. The DSC measurements were used for further investigation of the studied compounds. When a sample of Ph-C≡C-*dtpy* was heated, the endothermic peak due to melting was observed. However, both metal complexes melted with decomposition.

## 3. Materials and Methods

### 3.1. General Information

2-Acetylthiazole (98%, ABCR), phenylpropargyl aldehyde (96%, Merck-Sigma Aldrich, St. Louis, MO, USA), and Re(CO)_5_Cl (Merck-Sigma Aldrich) were commercially available and were used without further purification, while the precursor [Pt(PhCN)_2_Cl_2_] (99% purity) was obtained from Strem Chemicals Inc. (Newburyport, MA, USA). All solvents used for synthesis were of reagent grade and were used as received. For spectroscopy studies, HPLC grade solvents were used.

### 3.2. Preparation of Ligand Ph-C≡C-dtpy

In a conical flask closed with a glass stopper, 2-acetylthiazole (2.68 g, 21 mmol) and phenylpropargyl aldehyde (3-phenyl-2-propynal) (1.30 g, 10 mmol) were dissolved in 75 mL of ethyl alcohol. Potassium hydroxide (1.54 g, 27 mmol) and ammonia (35 mL) were then added to the flask in quick succession. The resulting reaction mixture was stirred for 24 h at room temperature. After this time, the precipitate was filtered off, washed thoroughly with water, and dried. The crude product was crystallized in methanol to obtain 4-phenylacetylene-2,6-bis(thiazol-2-yl)pyridine as a beige solid.

**Ph-C≡C-*dtpy*:** Yield: 18%. ^1^H NMR (500 MHz, CDCl_3_, 128 scans) δ 8.29 (s, 2H, H^B2^), 7.95 (d, *J* = 3.2 Hz, 2H, H^A1^), 7.59–7.55 (m, 2H, H^C4^), 7.49 (d, *J* = 3.1 Hz, 2H, H^A2^), 7.42–7.38 (m, 3H, H^C5,C6^). ^13^C NMR (125 MHz, CDCl_3_, 1024 scans) δ 168.23 (C^A3^), 151.33 (C^B1^), 144.40 (C^A1^), 134.11 (C^C3^), 132.22 (C^C4^), 129.57 (C^C6^), 128.70 (C^C5^), 122.17 (C^A2^), 122.11 (C^B3^), 121.97 (C^B2^), 95.31 (C^C2^), 86.56 (C^C1^).

### 3.3. Preparations of Re(I) Complex

Re(CO)_5_Cl (0.10 g, 0.27 mmol) and the Ph-C≡C-*dtpy* ligand (0.27 mmol) were dissolved in toluene (50 mL) and refluxed under argon for 8h. The resulting solution was allowed to cool to room temperature. X-ray quality, orange crystals were collected by filtration after a few days.

**[ReCl(CO)_3_(Ph-C≡C-*dtpy*)]:** Yield: 70%; C_22_H_11_ClN_3_O_3_S_2_Re: calcd. C 40.58, H 1.70, N 6.45% found: C 40.10, H 1.65, N 6.58%; ^1^H NMR (500 MHz, CDCl3, 128 scans): δ 8.24 (s, 1H), 8.19–8.12 (m, 2H), 7.89 (s, 1H), 7.79–7.75 (m, 2H), 7.64–7.61 (m, 2H), 7.51–7.44 (m, 3H); ^13^C NMR (125 MHz, CDCl_3_, 1024 scans) δ 195.70, 193.59, 188.98, 168.75, 163.53, 155.66, 152.47, 145.52, 144.16, 135.53, 132.38, 130.61, 130.38, 130.25, 129.19, 128.81, 127.84, 125.42, 123.78, 120.67, 100.98, 84.81; IR (KBr, cm^–1^): 2022(vs), 1919(vs), 1890(vs) ν(C=O); 1609(s), 1538(w) ν(C=N) and ν(C=C).

### 3.4. Preparations of Pt(II) Complex

A suspension of [Pt(PhCN)_2_Cl_2_] (0.24 g, 0.5 mmol) in acetonitrile (30 cm^3^) was treated with an equimolar amount of Ag(CF_3_SO_3_) (0.13 g, 0.5 mmol) dissolved in acetonitrile (10 cm^3^). The reaction mixture was heated under reflux for 16 h, and then the AgCl precipitate was removed by filtration and one equivalent of the ligand Ph-C≡C-*dtpy* was added to the filtrate. The reaction mixture was heated in a solvothermal reactor under atmospheric pressure for an additional 24 h and then gradually cooled for another 24 h. The crystals suitable for X-ray analysis were obtained directly from the mother liquor.

**[Pt(Ph-C≡C-*dtpy*)Cl]CF_3_SO_3_:** Yield: 55% C_19_H_11_ClN_3_S_2_Pt, CF_3_SO_3_: calcd. C 39.62, H 1.92, N 7.30%; found: 39.49, H 2.04, N 6.97% ^1^H NMR (500 MHz, DMSO, 128 scans) δ 8.83 (s, 2H), 8.52 (d, *J* = 3.4 Hz, 2H), 7.97 (d, *J* = 3.4 Hz, 2H), 7.73–7.71 (m, 2H), 7.63–7.57 (m, 3H).; ^13^C NMR (125 MHz, DMSO, 1024 scans) δ 169.58, 151.17, 141.53, 141.53, 136.48, 132.58, 131.51, 129.74, 125.44, 120.66, 100.72, 86.73; IR (KBr, cm^–1^): 1606(s), 1493(m), 1479 (m), 1443(m), 1262(vs), 1150(s), 1029(vs), 760(m), 691(m), 603(w), 636(s), 569(w), 515(w).

### 3.5. Crystal Structure Determination and Refinement

Single crystals of Ph-C≡C-*dtpy* and [ReCl(CO3)(Ph-C≡C-*dtpy*)] were obtained by the slow evaporation of toluene at room temperature. X-ray diffraction data were collected using an Oxford Diffraction Gemini A Ultra four-circle diffractometer equipped with an Atlas CCD detector (Agilent, Santa Clara, CA, USA) and graphite-monochromated MoKα radiation (λ = 0.71073 Å) at room temperature. Data were processed with the aid of CrysAlisPro software [48], Olex2 software [49], and the SHELXS and SHELXL-2014 packages [50]. All non-hydrogen atoms were refined anisotropically, while hydrogen atoms were placed in calculated positions and refined using riding constraints: d(C–H) = 0.93 Å, U_iso_(H) = 1.2 U_eq_(C) (for aromatic) and d(C–H) = 0.96 Å. Crystallographic data for Ph-C≡C-*dtpy* and [ReCl(CO_3_)(Ph-C≡C-*dtpy*)] have been deposited with the Cambridge Crystallographic Data Center (CCDC 2417181-2417182).

### 3.6. Computational Details

The calculations were performed using the GAUSSIAN-09 program package (C.01). The geometries of the singlet ground states (S_0_), and the lowest triplet states (T_1_) were fully optimized, without any symmetry restrictions at the DFT level, using the PBE1PBE hybrid exchange–correlation functional. The calculations were performed using the def2-TZVPD basis set for rhenium(I) and platinum(II) and the def2-TZVP basis set for the chlorine, oxygen, nitrogen, carbon, and hydrogen atoms. The starting point for geometry optimization was taken from the X-ray structure, and all the subsequent calculations were performed based on the optimized geometries. Vibrational frequencies were calculated on the basis of the optimized geometry to verify that each of the geometries corresponded to a minimum on the potential energy surface. Furthermore, on the basis of the optimized ground and excited state geometries, the absorption and emission properties in acetonitrile (CH_3_CN) media were calculated by TD-DFT at the PBE1PBE hybrid functional level and with the polarized continuum model (PCM). The predicted bond lengths of the free ligand and Re(I) complex are compared with the experimental data in Table S2. The predicted bond lengths and angles for the ground state are within the range of error expected for DFT calculations of rhenium(I) complexes, and the general trends observed in the experimental data are well reproduced in the calculations, providing confidence in the reliability of the chosen method to reproduce the geometry of the studied complex [40,51].

### 3.7. Physical Measurements

IR spectra were recorded using a Nicolet iS5 FT-IR spectrophotometer (Thermo Fisher Scientific, Santa Clara, CA, USA) in the range of 4000–400 cm^−1^ with KBr pellets. Electronic absorption spectra were measured on a Nicolet Evolution UV-VIS-NIR spectrophotometer in the range of 800–250 nm in solution (c = 2.5 × 10^−5^ mol dm^−3^). For the evaluation of stability in three solvents, CHCl_3_, CH_3_CN, and DMSO, the UV-Vis spectra (250–800 nm) of the complexes were recorded after 5 h using a NanoDrop One/One Microvolume UV-Vis Spectrophotometer (Thermo Fisher Scientific). The ^1^H and ^13^C NMR spectra were recorded at 295 K in dimethyl sulfoxide-d^6^ on a Bruker Avance 400 MHz NMR (Billerica, MA, USA).

Cyclic voltammetry (CV) and differential pulse voltammetry (DPV) measurements were carried out on the Autolab potentiostat (Eco Chemie, Utrecht, The Netherlands). A three-electrode, one-compartment cell was used to contain the solution of complexes and supporting electrolyte in DMF. A glass carbon disk as the working electrode, a silver wire as the quasi-reference electrode, a platinum coil as the auxiliary electrode, and ferrocene as the standard were used during measurements. Deaeration of the solution was achieved by argon bubbling through the solution for about 20 min before measurement. The complexes and supporting electrolyte (n-Bu_4_NPF_6_) concentrations were equal to 10^−6^ mol dm^−3^ and 0.01 mol dm^−3^, respectively. The scan rate was equal to 0.1 V s^−1^. A glassy carbon disk working electrode (3 mm diam.) and an Ag/Ag^+^ reference electrode were used. All electrochemical experiments were carried out under ambient conditions. Thermogravimetric analysis (TGA) was carried out on a Discovery TGA 55-TA Instruments Thermal Analyzer (New Castle, DE, USA) with 10 °C min^−1^ in a stream of nitrogen (20 cm^3^ min^−1^) in the temperature range of 25–800 °C. Differential Scanning Calorimetry (DSC) was performed with a DSC 25-TA Instruments Thermal Analyzers apparatus (New Castle, DE, USA), under a nitrogen atmosphere(50 cm^3^ min^−1^) and using sealed aluminum pans with a heating rate of 10 °C min^−1^.

### 3.8. Photoluminescence Spectra

Photoluminescence spectra were recorded using an FLS-980 spectrofluorometer (Edinburgh, Livingston UK). Room-temperature measurements were conducted in DMSO (c = 2.5 × 10^−5^ mol·dm^−3^). Low-temperature emission spectra were obtained in a frozen-glass matrix of an ethanol/methanol mixture (4:1) at liquid nitrogen temperature using a Dewar assembly. The time-resolved measurements were carried out in optically diluted (0.05 < O.D < 0.1) solutions at room temperature using the time-correlated single photon counting (TCSPC) methods on the FLS-980 spectrofluorometer. The excitation wavelength was obtained using the set of picosecond pulsed diodes (EPLED–340 nm, EPLED–375 nm) as the light source, and PMT (Hamamatsu, Shizuoka, Japan, R928P) in cooled housing was used as a detector. The system was aligned at the emission wavelength. Additionally, for the analysis of fluorescence decay, an instrument response function (IRF) was obtained. The IRF contains information about the time response of the overall optical and electronic system. The IRF was designated using ludox solution as a standard at excitation wavelengths. Quantum yields were measured using the integrating sphere absolute method at room temperature and the solvent as a blank. The samples were optically diluted to avoid the inner filter effect and re-absorption.

## 4. Conclusions

Over the last decades, *terpys* have been successfully utilized for the synthesis of d-block metal compounds, owing to their rich and often complex photophysical properties. In last years, *dtpy* derivatives have also emerged as promising ligands for the preparation of transition metal complexes, in which free ligand binds to the Re(I) and Pt(II) centres in a bidentate or tridentate bonding mode, respectively. It is worth noting that the incorporation of a 4-phenylacetylene substituent into a *dtpy* skeleton has been explored for the first time in synthesis platinum(II) and tricarbonyl rhenium(I) compounds. Most remarkably, the introduction of this substituent led to the observation of an MLCT transition in a Re(I) complex, while an ILCT transition dominated in the case of the Pt(II) complex. Both complexes exhibited long-lived emissions at room temperature, with the Re(I) demonstrating a higher quantum yield and longer wavelength emission. Lowering the temperature from 298 K to 77 K results in an increased excited state lifetime in both systems, together with a blue shift in their respective emission maxima. The studied compounds show two reversible reduction waves without any oxidation process observed. Thermal studies showed that Pt(II) is characterized by the highest T_5%_ and T_10%_, while Re(I) has the highest T_max_. In the near future, studies on the luminescence properties of 4-R-acetylene-*dtpy* (R-phenyl, naphthalenyl, anthryl) toward its metal complexes will be continued.

## Figures and Tables

**Table 1:** Photoluminescence properties for free ligand and metal complexes.

	Medium	Excitation	Emission	Φ	τ [ns]	τ_eff_ [ns]	χ^2^
Ph-C≡C-*dtpy*	CHCl_3_	338, 307	385	21.27	1.58	1.58	1.071
	CH_3_CN	337, 314, 272	386	18.83	1.50	1.50	1.003
	solid	467	564	5.10	7.94 (47.1%) 1.89 (52.9%)	4.74	1.278
	77 K	345, 316	388, 370	–	2.92	2.92	1.056
[ReCl(CO)_3_(Ph-C≡C-*dtpy*)]	CHCl_3_	470 (sh), 362, 308, 261	720	8.39	4.33 (30.8%) 2.42 (69.20%)	3.01	0.894
	CH_3_CN	no stable					
	solid	527	655	6.43	1298.7 (75.8%) 151.86 (24.2%)	1021.16	1.046
	77 K	395, 355, 323	600	–	36,014.47 (20.8%) 1652.15 (79.2%)	8799.51	1.136
[Pt(Ph-C≡C-*dtpy*)Cl]CF_3_SO_3_	CHCl_3_	insoluble					
	CH_3_CN	380, 444	580, 620	1.91	9.29 (6.0%) 559.94 (59.3%) 2869.88 (34.7%)	1328.45	1.078
	solid	540, 600, 647	700	8.53	43.49 (19.0%) 133.34 (51.3%) 495.96 (29.7%)	223.97	1.117
	77 K	420, 347, 306	553, 600, 653	–	4291.06 (68.3%) 27,598.21 (31.7%)	11,679.43	1.060

**Table 2:** Electrochemical data.

Compound	$E_{pa}^{red1}$	$E_{pc}^{red1}$	ΔE	$E_{\frac{1}{2}}$	${Red}_{onset}^{1}$	${Red}_{onset}^{2}$	E_A_(LUMO)	* IP(HOMO) ^a^	Eg_(opt)_^g^
Ph-C≡C-*dtpy*	−2.00	−2.07	0.07	−2.04	−1.95	−2.47	−3.15	−6.49	3.34
[ReCl(CO)_3_(Ph-C≡C-*dtpy*)]	−1.38	−1.47	0.09	−1.42	−1.34	−1.84	−3.76	−6.22	2.46
[Pt(Ph-C≡C-*dtpy*)Cl]CF_3_SO_3_	−0.95	−1.03	0.08	−0.99	−0.91	−1.49	−4.19	−6.92	2.73

^a^ IP—the ionization potential estimated from the equation * IP = E_A_ − E_g(opt)_; ^g^ Eg_(opt)_ = 1241/λem; *E*_pa_ and *E*_pc_—the anodic and cathodic potentials vs. Fc/Fc^+^; Δ*E*—the peak potential difference estimated from the equation Δ*E* = *E*_pa_ − *E*_pc_; *E*_1/2_—the redox potentials estimated from the equation *E*_1/2_ = (*E*_pa_ + *E*_pc_)/2; *E*^ox1^_ons_—the oxidation onset potential; *E*^red1^_ons_—the reduction onset potential; IP—the ionization potential estimated from the equation; EA—the electron affinity estimated from the equation EA = |*e*^−^|(5.1 + *E*^red1^_ons_).

**Table 3:** Electrochemical literature data for selected Re(I) complexes.

General Formula	Type of R-Substituent	E_red1_[V]	E_red2_[V]	E_red3_[V]	Eg(opt)	Ref
[ReCl(CO)_3_(4-R-*dtpy*)]	4-pyridyl	–1.36	−1.85		2.18	[5]
	3-pyridyl	–1.57	−1.96		2.18	
	thiophen-5-yl	–1.60	−1.97		2.32	
	2,2′-bithiophen-5-yl	−1.61	−2.01		1.86	
	furanyl	−1.65	−2.05		2.31	
	furan and vinyl bond	−1.58	−2.00		1.89	
	N-methyl-pyrrol-2-yl	−1.74	−2.16		2.25	
	carbazolyl	–1.64	−2.04		2.29	
	phenyl	−1.60	−2.00		2.26	[6]
	pyrrolidine	−1.71	−2.10		2.03	[6]
[Pt(4-R-*dtpy*)Cl]CF_3_SO_3_	9-anthryl	−1.02	−1.48	-		[21]
	9-phenanthryl	−1.00	−1.59	−2.14		
	1-pyrenyl	−0.95	−1.56	−1.77		

## Data Availability

Data are contained within the article and Supplementary Materials.

## Supplementary Materials

The following supporting information can be downloaded at https://www.mdpi.com/article/10.3390/molecules30040915/s1. Figure S1: IR spectra for ligand and metal complexes together with short comparison of experimental and calculated most important vibrations in the infrared spectrum; Figure S2: ^1^H NMR and ^13^C NMR spectra for ligand and metal complexes; Figure S3: Composition of frontier molecular orbitals of Re(I) and Pt(II) complexes; Figure S4: The contours of the frontier molecular orbitals; Figure S5: UV-Vis absorption spectra of [ReCl(CO)_3_(Ph-C≡C-*dtpy*)] in CH_3_CN recorded once every 25 min for 5 h; Figure S6: Luminescent properties of studied compounds in solid-state, low-temperature glass matrix (EtOH:MeOH, 4:1 *v*/*v*), acetonitrile and chloroform solutions. Lifetime decay curves were measured using EPL–405 picosecond pulsed diode laser (Edinburgh Instruments) λ_Ex_ = 405 nm; Figure S7: Cyclic voltammograms for free ligand and transition metal compounds. Table S1. Crystal data and structure refinement; Table S2. Selected bond lengths (Å) and angles (deg) for 1 and 6; Table S3. Short intra–and intermolecular contacts; Table S4. Short π•••π stacking interactions; Table S5. Short C–H•••π stacking interactions; Table S6. The energies and characters of the selected spin-allowed electronic transitions for Ph-C≡C-*dtpy*, together with assignment to the experimental absorption bands; Table S7. The energies and characters of the selected spin-allowed electronic transitions for [ReCl(CO)_3_(Ph-C≡C-*dtpy*)], together with assignment to the experimental absorption bands; Table S8. The energies and characters of the selected spin-allowed electronic transitions for [Pt(Ph-C≡C-*dtpy*)Cl]CF_3_SO_3_, together with assignment to the experimental absorption bands; Table S9. TGA data; Table S10. Photophysical data for free *dtpy*-like ligands; Table S11. Photophysical data for tricarbonyl Re(I) compounds based on *dtpy*-like ligands; Table S12. Photophysical data for Pt(II) complexes based on *dtpy*-like ligands. refs [5,6,20,23,24,25,37,52,53,54,55] are cited in Supplementary Materials.
