# Supplementary material for: Acetylenic Substituent: Influence on the Structure, Electrochemical, Photophysical, and Thermal Properties of Rhenium(I) and Platinum(II) Complexes
Source: Molecules. 2025 Feb 16;30(4):915. doi: 10.3390/molecules30040915 (PMC11858454; doi:10.3390/molecules30040915)
Supplement: Supplementary file 1 [file molecules-30-00915-s001.zip › molecules-3479423-supplementary.pdf]

Acetylenic substituent: Influence on the structure, electrochemical, photophysical and thermal properties of rhenium(I) and platinum(II) complexes

Bartosz Zowiślok , Anna Świtlicka*, Anna Maria Maroń* and Sławomir Kula

Institute of Chemistry, University of Silesia, 9 Szkolna Str., 40-006 Katowice, Poland

Corresponding authors: anna.switlicka@us.edu.pl, anna.maron@us.edu.pl

Figure S1. IR spectra for ligand and metal complexes together with short comparison of experimental and calculated the most important vibrations in the infrared spectrum 4

Figure S2. ^{1}H NMR and ^{13}C NMR spectra for ligand and metal complexes 8

Table S1. Crystal data and structure refinement.. 9

Table S2. Selected bond lengths (Å) and angles (deg) for Ph-C≡C-dtpy and [ReCl(CO)$_3$(Ph-C≡C-dtpy)] 10

Table S3. Short intra–and intermolecular contacts 10

Table S4. Short π•••π stacking interactions... 11

Table S5. Short C–H•••π stacking interactions 11

Table S6. The energies and characters of the selected spin-allowed electronic transitions for Ph-C≡C-dtpy, together with assignment to the experimental absorption bands.................. 12

b) chloroform... 12

Table S7. The energies and characters of the selected spin-allowed electronic transitions for [ReCl(CO)$_3$(Ph-C≡C-dtpy)], together with assignment to the experimental absorption bands (chloroform).. 13

Table S8. The energies and characters of the selected spin-allowed electronic transitions for [Pt(Ph-C≡C-dtpy)Cl]CF$_3$SO$_3$, together with assignment to the experimental absorption bands (acetonitrile).. 14

Figure S3. Composition of frontier molecular orbitals of Re(I) and Pt(II) complexes 15

Figure S4. UV-Vis absorption spectra of [ReCl(CO)$_3$(Ph-C≡C-dtpy)] in different solvents recorded once every 25 minutes for 5 h 16

Figure S5. The contours of the frontier molecular orbitals 17

Figure S6. Luminescent properties of studied compounds in solid state, low temperature glass matrix (EtOH:MeOH, 4:1 v/v), acetonitrile and chloroform solutions. The lifetime decay curves were measured using EPL – 375 picosecond pulsed diode laser (Edinburgh Instruments), λ_{Ex} = 405nm 18

Table S9. TGA and DSC data 20

Figure S7. Cyclic voltammograms for free ligand and transition metal compounds........... 21

Table S10. Photophysical data for free dtpy-like ligands 22

Table S11.Photophysical data for tricarbonyl Re(I) compounds based on dtpy-like ligands 26

Table S12.Photophysical data for Pt(II) complexes based on dtpy-like ligands.................... 31

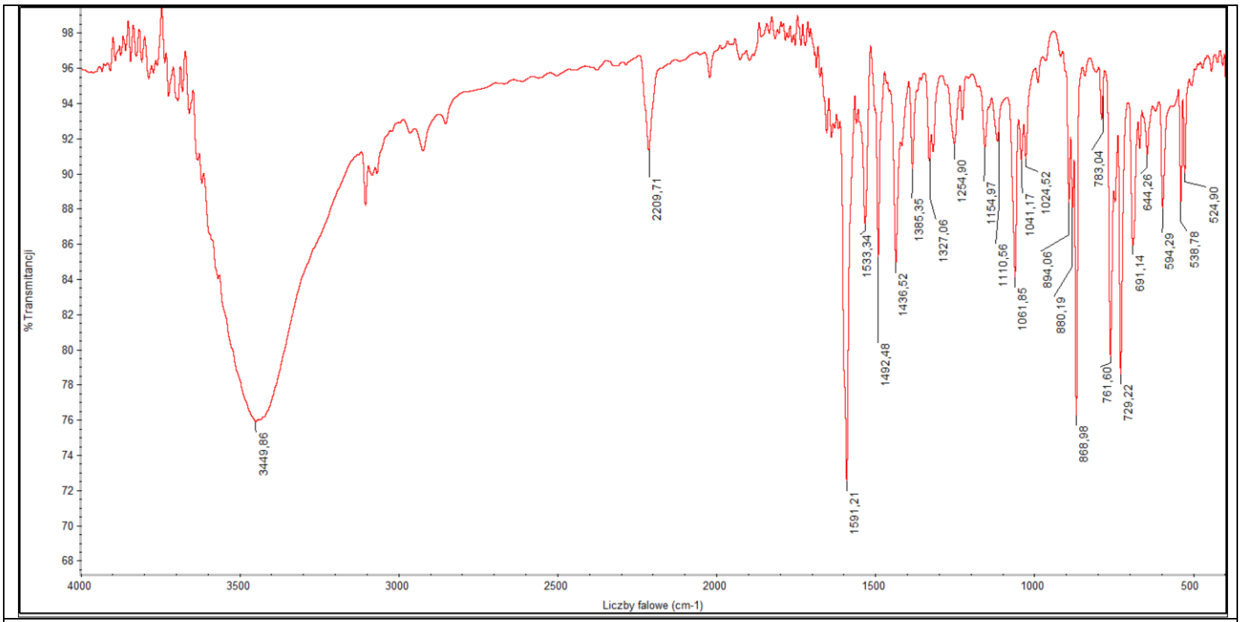

Ph-C≡C-dtpy

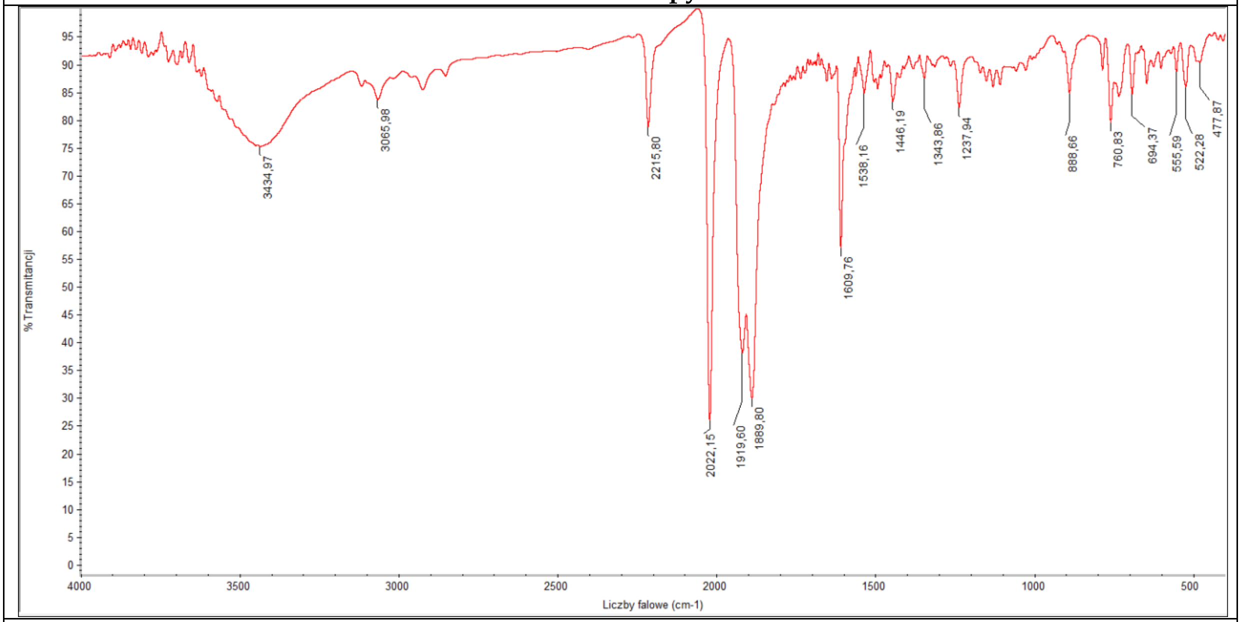

[ReCl(CO_3)(Ph-C≡C-dtpy)]

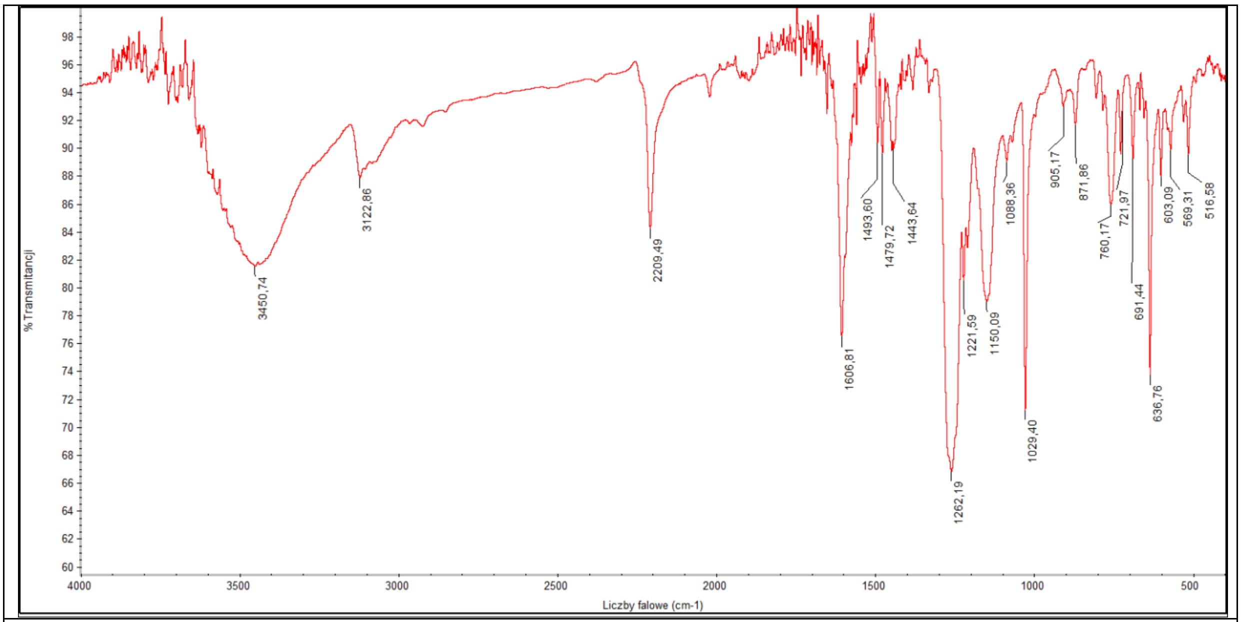

[Pt(Ph-C≡C-dtpy)Cl]CF$_3$SO$_3$

	**Experimental**	**Calculated**
Ph-C≡C-dtpy	$\nu_{C\equiv C}$ = 2209.71 cm^{-1}<br>$\nu_{C=C,C=N}$ = 1591.21 cm^{-1}	$\nu_{C\equiv C}$ = 2323 cm^{-1}<br>$\nu_{C=C,C=N}$ = 1644 cm^{-1},<br>1603. cm^{-1}
[Pt(Ph-C≡C-dtpy)Cl]CF$_3$SO$_3$	$\nu_{C\equiv C}$ = 2209.49 cm^{-1}<br>$\nu_{C=C,C=N}$ = 1606.81 cm^{-1}, -	$\nu_{C\equiv C}$ = 2313 cm^{-1}<br>$\nu_{C=C,C=N}$ = 1669 cm^{-1}, 1656 cm^{-1}
[ReCl(CO)$_3$(Ph-C≡C-dtpy)]	$\nu_{C\equiv C}$ = 2215cm^{-1}<br>$\nu_{C=C,C=N}$ = 1609 cm^{-1}, 1538 cm^{-1}<br>$\nu_{C\equiv O}$=2022 cm^{-1}, 1919 cm^{-1}, 1889 cm^{-1}	$\nu_{C\equiv C}$ = 2317 cm^{-1}<br>$\nu_{C=C,C=N}$ = 1672 cm^{-1}, 1551 cm^{-1}<br>$\nu_{C\equiv O}$=2099 cm^{-1}, 1962 cm^{-1},<br>1986 cm^{-1}

Figure S1. IR spectra for ligand and metal complexes together with short comparison of experimental and calculated the most important vibrations in the infrared spectrum

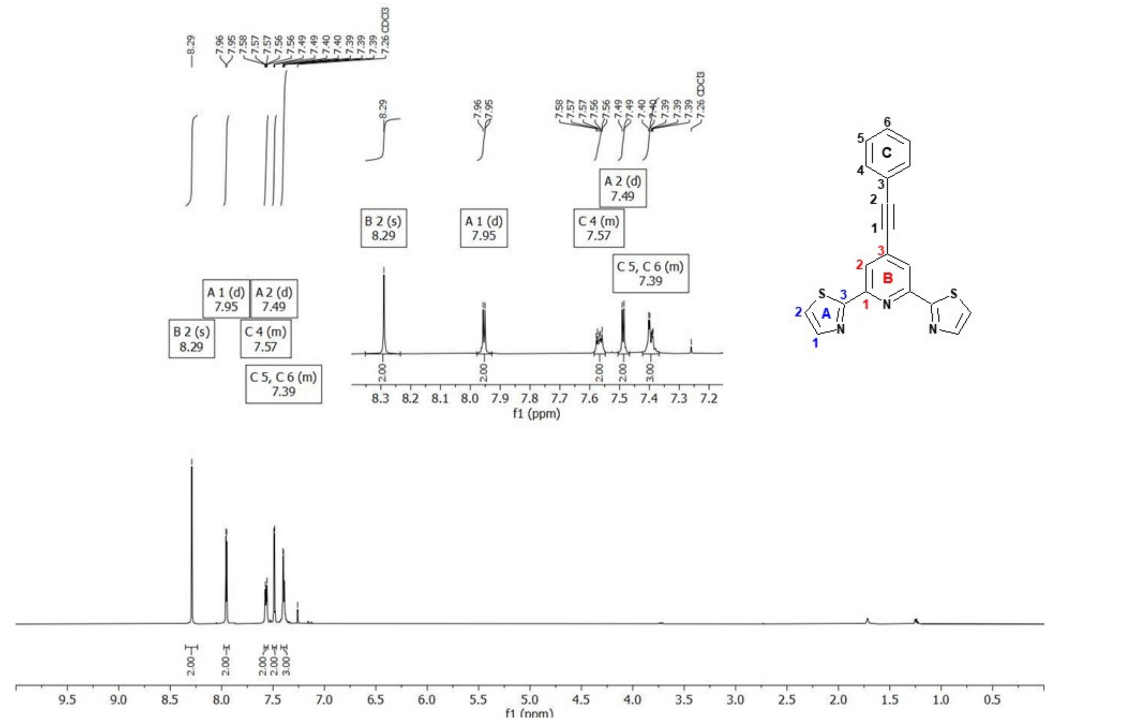

¹H NMR for Ph-C≡C-dtpy

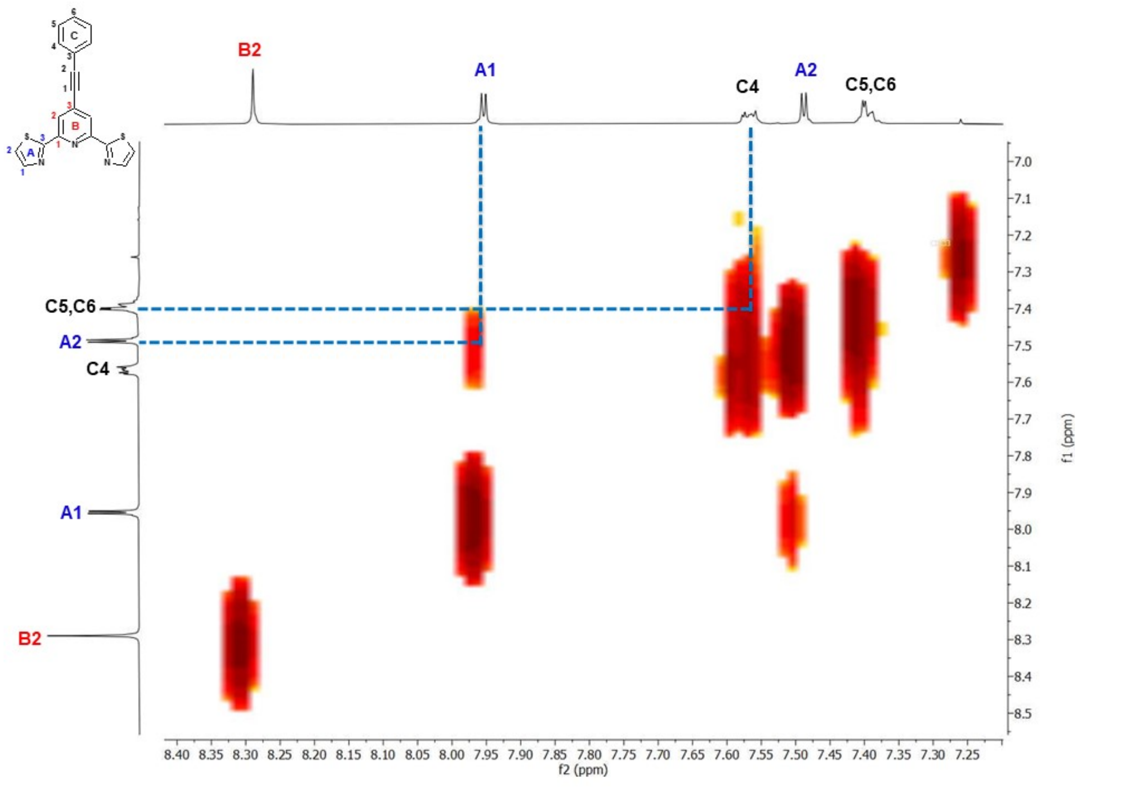

¹H-¹H COSY for Ph-C≡C-dtpy

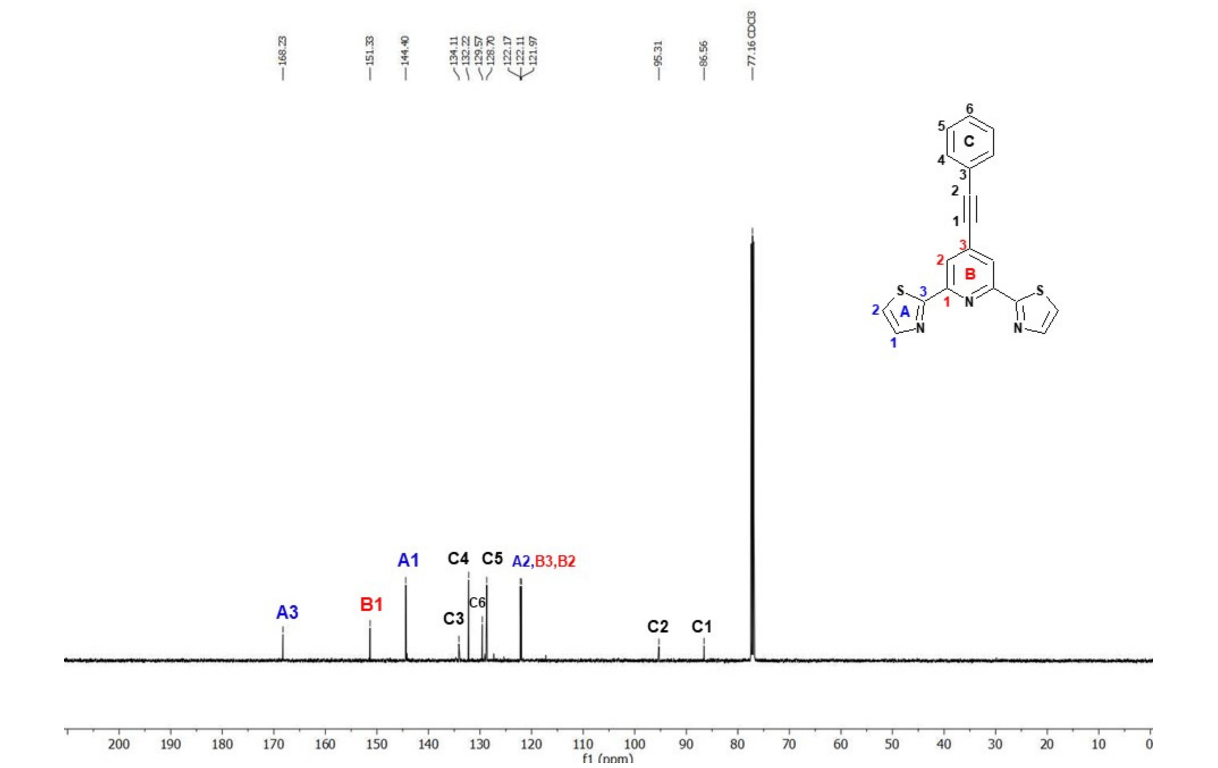

^{13}C NMR for Ph-C≡C-dtpy

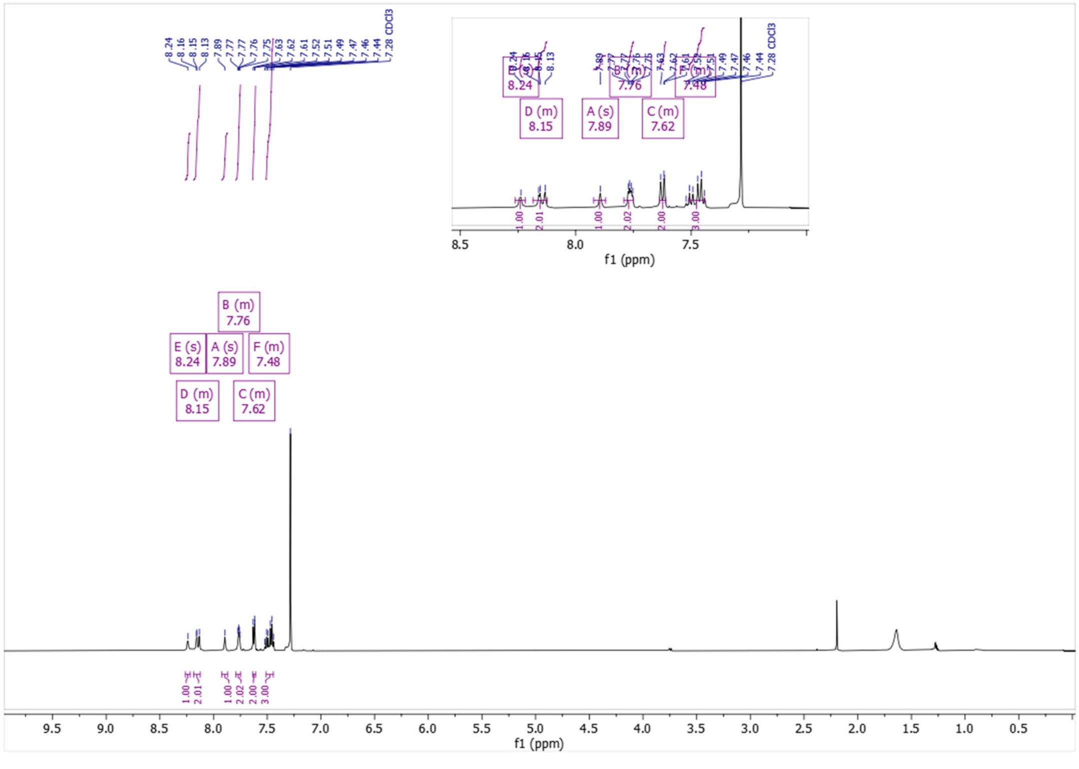

^{1}H NMR for [ReCl(CO)$_3$(Ph-C≡C-dtpy)]

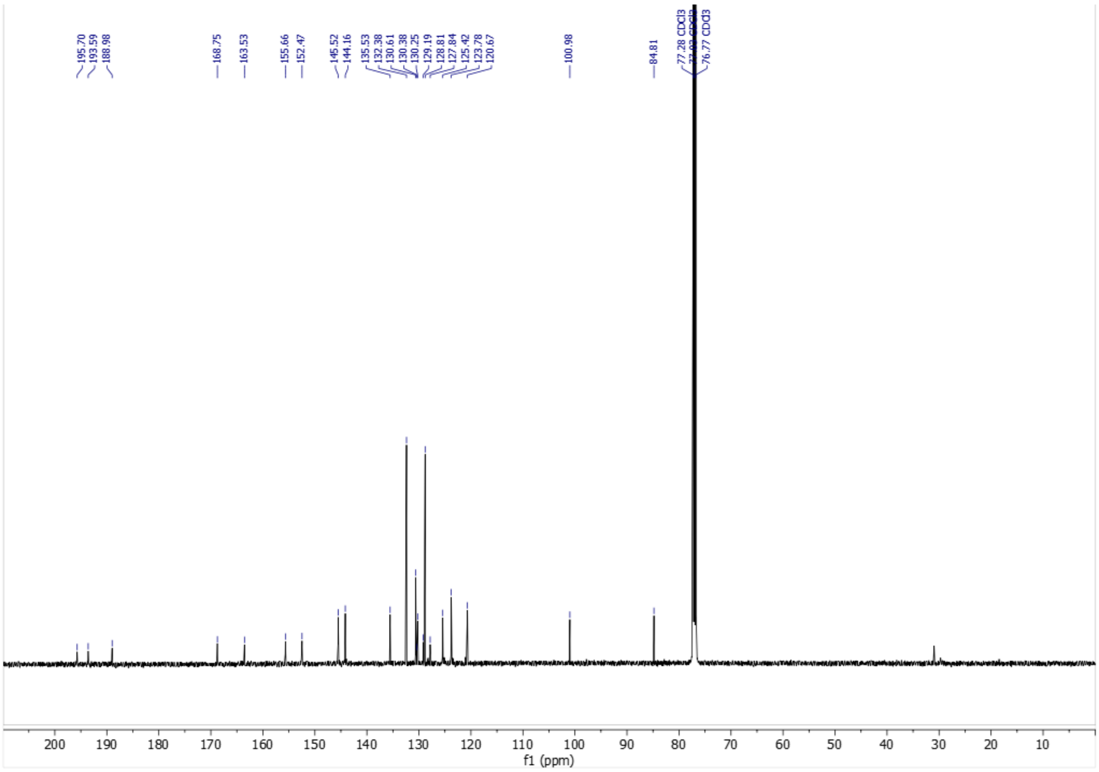

^{13}C NMR for [ReCl(CO)$_3$(Ph-C≡C-dtpy)]

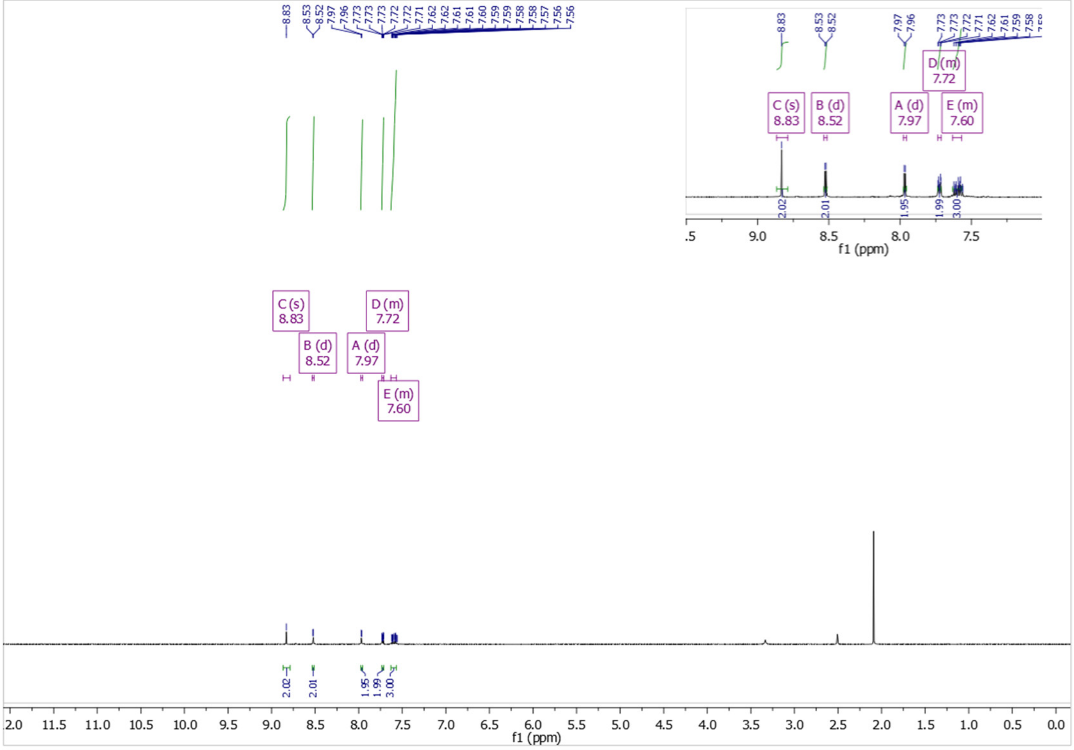

^{1}H NMR for [Pt(Ph-C≡C-dtpy)Cl]CF_3SO_3

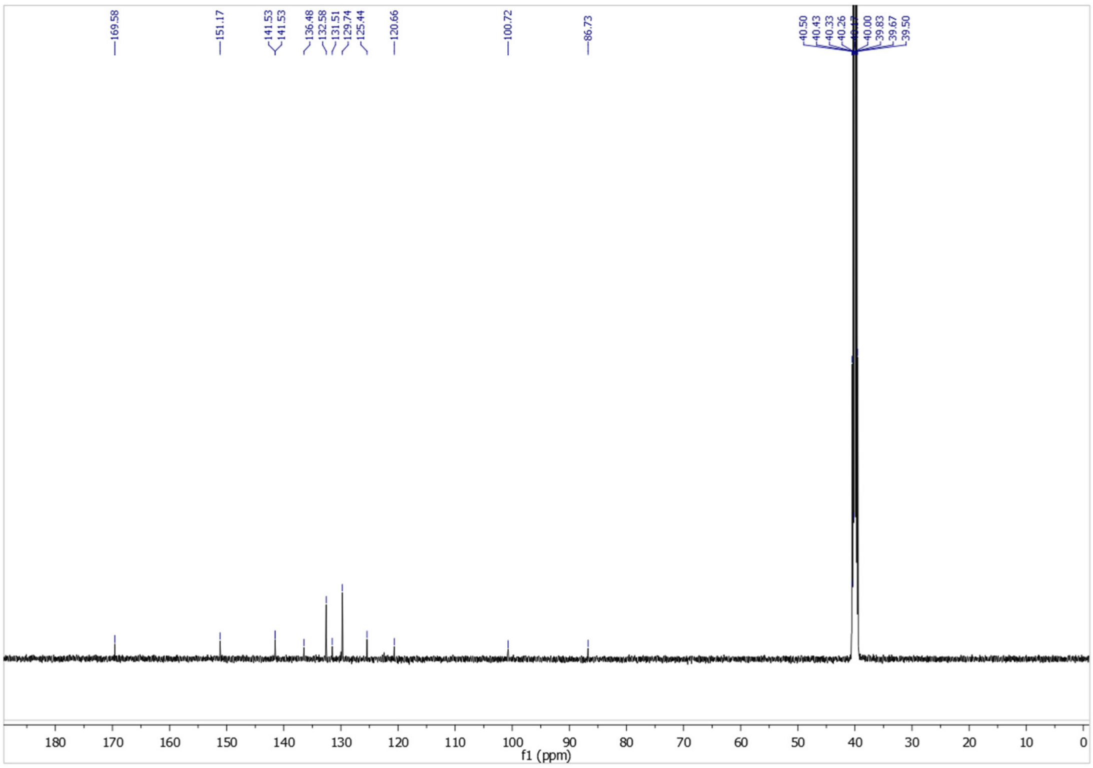

^{13}C NMR for [Pt(Ph-C≡C-dtpy)Cl]CF_3SO_3

Figure S2. ^{1}H NMR and ^{13}C NMR spectra for ligand and metal complexes

Table S1. Crystal data and structure refinement

	**Ph-C≡C-dtpy**	**[ReCl(CO$_3$)(Ph-C≡C-dtpy)]**
Empirical formula	$C_{19}H_{11}N_3S_2$	$C_{22}H_{10}ClN_3O_3S_2Re$
Formula weight	345.43	650.10
Temperature [K]	295.0(2)	295.0(2)
Wavelength [Å]	0.71073	0.71073
Crystal system	monoclinic	monoclinic
Space group	$P2_1/c$	$P2_1/n$
Unit cell dimensions [Å, °]	a = 5.7796(3)<br>b = 14.4947(9)<br>c = 19.4995(13)<br><br>β = 91.368(6)	a =11.2787(6)<br>b = 15.6473(8)<br>c = 13.6119(9)<br><br>β = 106.832(6)
Volume [Å^3]	1633.09(18)	2299.3(2)
Z	4	4
Density (calculated) [Mg/m^3]	1.405	1.878
Absorption coefficient[mm^{-1}]	0.330	5.612
F(000)	712	1244
Crystal size [mm]	0.25 x 0.21 x 0.18	0.21 x 0.18 x 0.12
θ range for data collection [°]	3.50 to 25.05	to 25.05
Index ranges	$-7 \leq h \leq 7$<br>$-19 \leq k \leq 16$<br>$-26 \leq l \leq 20$	$-14 \leq h \leq 15$<br>$-21 \leq k \leq 19$<br>$-18 \leq l \leq 17$
Reflections collected	8946	16391
Independent reflections	3916 (R_{int}=0.0287)	5574 (R_{int}= 0.0372)
Completeness to 2θ [%]	99.8	99.7
Max. and min. transmission	1.00 and 0.772	1.00 and 0.333
Data / restraints / parameters	3916/ 0 / 217	5574 /0 / 298
Goodness-of-fit on F^2	1.027	1.059
Final R indices [$I > 2\sigma(I)$]	*R1* = 0.0483<br>*wR2* =0.0961	*R1* = 0.0365<br>*wR2* =0.0659
R indices (all data)	*R1* = 0.0851<br>*wR2* = 0.1173	*R1* = 0.0609<br>*wR2* =0.0761
Largest diff. peak and hole[eÅ^{-3}]	0.228 and -0.208	1.289 and -0.652

Table S2. Selected bond lengths (Å) and angles (deg) for Ph-C≡C-dtpy and [ReCl(CO)$_3$(Ph-C≡C-dtpy)]

**Bond lengths [Å]**			**Bond angles [°]**		
			**Ph-C≡C-dtpy**		
	experimental	calculated		experimental	calculated
S(1)–C(1)	1.697(3)	1.70214	C(1)–S(1)–C(3)	89.02(11)	89.440
S(1)–C(3)	1.722(2)	1.72332	C(11)–S(2)–C(9)	88.90(13)	89.440
S(2)–C(9)	1.716(2)	1.72332	C(8)–N(1)–C(4)	117.52(17)	118.374
S(2)–C(11)	1.699(3)	1.70214	C(3)–N(2)–C(2)	110.4(2)	110.864
N(1)–C(4)	1.341(3)	1.33119	C(9)–N(3)–C(10)	109.8(2)	110.864
N(1)–C(8)	1.329(3)	1.33119			
N(2)–C(2)	1.360(3)	1.35920			
N(2)–C(3)	1.305(3)	1.30404			
N(3)–C(9)	1.296(3)	1.30404			
N(3)–C(10)	1.365(3)	1.35921			
			**[ReCl(CO$_3$)(Ph-C≡C-dtpy)]**		
Re(1)–N(1)	2.167(4)	2.16817	N(1)–Re(1)–N(2)	74.79(14)	74.338
Re(1)–N(2)	2.236(4)	2.26404	N(1)–Re(1)–C(1)	175.37(18)	17.336
Re(1)–C(1)	1.900(6)	1.90407	N(1)–Re(1)–C(2)	98.2(2)	96.772
Re(1)–C(2)	1.903(6)	1.92242	N(1)–Re(1)–C(3)	93.88(18)	94.197
Re(1)–C(3)	1.903(5)	1.90462	N(1)–Re(1)–Cl(1)	84.48(9)	84.205
Re(1)–Cl(1)	2.4857(11)	2.49570	N(2)–Re(1)–C(1)	101.71(18)	101.876
			N(2)–Re(1)–C(2)	171.61(18)	169.941
			N(2)–Re(1)–C(3)	95.75(18)	96.989
			N(2)–Re(1)–Cl(1)	81.52(9)	81.745
			C(1)–Re(1)–C(2)	85.1(2)	86.623
			C(3)–Re(1)–C(2)	89.2(2)	88.228
			C(1)–Re(1)–C(3)	89.5(2)	90.429
			C(1)–Re(1)–Cl(1)	92.04(16)	91.117
			C(2)–Re(1)–Cl(1)	93.38(16)	92.829
			C(1)–Re(1)–Cl(3)	177.10(16)	178.176

Table S3. Short intra–and intermolecular contacts

**D−H•••A**	**D−H**	**H•••A**	**D•••A[Å]**	**D−H•••A[°]**
		**[ReCl(CO$_3$)(Ph-C≡C-dtpy)]**		
C(5)–H(5)•••Cl(1)[a]	0.93	2.73	3.563(5)	150.00

Symmetry codes: (a): 3/2-*x*,-1/2+*y*,3/2-*z*

Table S4. Short π•••π stacking interactions

Cg(I)•••Cg(J)	Cg(I)•••Cg(J) [Å]	α [°]	β [°]	γ [°]	Cg(I)-Perp [Å]	Cg(J)-Perp [Å]
			**Ph-C≡C-dtpy**			
Cg(1)•••Cg(2)[b]	3.8480(13)	4.68(11)	20.82	25.49	3.4735(10)	-3.5967(8)

*α = dihedral angle betweenCg(I) andCg(J);Cg(I)-Perp = Perpendicular distance ofCg(I) on ring J;Cg(J)-Perp = perpendicular distance ofCg(J) on ring I; β = angleCg(I)→Cg(J) vector and normal to ring I; γ = angleCg(I)→Cg(J) vector and normal to plane J;
&Symmetrycode: (b) = -1+*x*, *y*, *z*
Cg(1) is the centroid of the S(2)/C(9)/N(3)/C(10)/C(11)
Cg(2) is the centroid of the N(1)/C(4)/C(5)/C(6)/C(7)/C(8)

Table S5. Short C–H•••π stacking interactions

Cg(I)•••Cg(J)	H•••Cg(J) [Å]	X-H•••Cg [Å]	X•••Cg [Å]
	**[ReCl(CO₃)(Ph-C≡C-dtpy)]**		
C(20)–H(20)•••Cg(1)[c]	2.94	3.607(11)	130

*α = dihedral angle betweenCg(I) andCg(J);Cg(I)-Perp = Perpendicular distance ofCg(I) on ring J;Cg(J)-Perp = perpendicular distance ofCg(J) on ring I; β = angleCg(I)→Cg(J) vector and normal to ring I; γ = angleCg(I)→Cg(J) vector and normal to plane J;
&Symmetry code: (c) = 1-x,1-y,-z; Cg(3):S2/C12/N3/C13/C14 is the centroid of the

Table S6. The energies and characters of the selected spin-allowed electronic transitions for **Ph-C≡C-dtpy**, together with assignment to the experimental absorption bands

a) acetonitrile

Experimental Absorption [nm]	Major contribution	Character	Energy (eV)	Wavelength [mn]	Osc. strength
338 nm	HOMO→LUMO (92%)	IL	2.98	343.97	*0.1946*
	H-1→LUMO (90%)		3.20	320.23	*1.1988*
	H-1→L+1 (86%)		3.45	296.87	*0.1638*
	HOMO→L+1 (92%)		3.45	296.83	*0.2278*
	H-2→LUMO (95%)		3.52	290.76	*0.032*
	H-2→L+1 (93%)		3.71	275.74	*0.1703*
	HOMO→L+2 (95%)		3.98	257.37	*0.0372*
	H-1→L+2 (93%)		4.09	250.57	*0.2137*
230 nm	H-6→LUMO (95%)		4.34	235.94	*0.118*
	H-3→L+2 (22%)<br>H-1→L+3 (54%)		4.43	231.28	*0.0193*
	H-2→L+2 (73%)		4.51	227.10	*0.0144*
	H-8→L+1 (91%)		4.70	217.86	*0.032*
	H-3→L+2 (66%)		4.98	205.47	*0.129*
	H-11→L+1 (58%)		5.25	195.16	*0.1068*

b) chloroform

Experimental Absorption (nm)	Major contribution	Character	Energy (eV)	Wavelength (nm)	*Osc. strength*
342	HOMO→LUMO (92%)	IL	2.98	344.00	0.2036
	H-1→LUMO (90%)		3.19	320.95	1.2324
310	HOMO→L+1 (91%)		3.44	297.20	0.2330
	H-1→L+1 (86%)		3.45	296.82	0.1709
	H-2→LUMO (95%)		3.53	290.34	0.0278
	H-2→L+1 (92%)		3.72	275.53	0.1698
	H-3→LUMO (80%),<br>H-1→L+3 (13%)		3.85	265.81	0.0099
	HOMO→L+2 (95%)		3.98	257.57	0.0362
	H-1→L+2 (93%)		4.08	250.89	0.2106
	H-6→LUMO (95%)		4.34	235.92	0.1219

Table S7. The energies and characters of the selected spin-allowed electronic transitions for **[ReCl(CO)$_3$(Ph-C≡C-dtpy)]**, together with assignment to the experimental absorption bands (chloroform)

**Experimental Absorption (nm)**	**Major contribution**	**Character**	**Energy (eV)**	**Wavelength (nm)**	***Osc. strength***
420	HOMO→LUMO (98%)	MLCT/ILCT	2.04	501.03	0.0043
	H-1→LUMO (96%)	MLCT/ILCT	2.22	462.21	0.1637
	H-2→LUMO (94%)	MLCT/ILCT	2.48	412.21	0.0192
	HOMO→L+1 (97%)	MLCT/ILCT	2.59	394.69	0.0261
310	H-1→L+1 (97%)	MLCT/ILCT	2.70	379.16	0.0276
	H-3→LUMO (89%)	ILCT/MLCT	2.89	354.15	0.7611
	H-4→LUMO (78%), H-2→L+1 (10%)	ILCT	2.95	347.58	0.2163
	H-5→LUMO (57%), H-3→L+1 (32%)	IL	3.28	312.08	0.2681
	H-7→LUMO (82%)	ILCT	3.43	298.47	0.0328
	H-1→L+2 (66%), H-1→L+3 (14%), H-1→L+5 (13%)	ILCT/IL IL	3.49	293.18	0.0212
	H-6→LUMO (22%), H-4→L+1 (55%)	IL	3.53	290.19	0.0513
270	H-10→LUMO (21%), H-9→LUMO (17%), H-8→LUMO (39%)	IL	3.62	282.70	0.0264
	H-5→L+1 (87%)	ILCT/IL	3.78	271.10	0.0828
	H-9→L+1 (29%), H-3→L+2 (43%)	ILCT/IL	4.12	248.70	0.0892
	H-4→L+2 (76%)	ILCT/IL	4.26	240.41	0.1082

Table S8. The energies and characters of the selected spin-allowed electronic transitions for **[Pt(Ph-C≡C-dtpy)Cl]CF_3SO_3**, together with assignment to the experimental absorption bands (acetonitrile)

Experimental Absorption (nm)	Major contribution	Character	Energy (eV)	Wavelength (nm)	Osc. strength
420	HOMO→LUMO (92%)	ILCT/MLCT	2.38	429.67	*0.4986*
	H-1→LUMO (92%)	ILCT/MLCT	2.47	415.19	*0.0108*
	HOMO→L+1 (88%)	ILCT/MLCT	2.64	387.46	*0.0212*
346	H-2→LUMO (81%)	ILCT/MLCT	2.83	362.45	*0.3368*
	H-1→L+1 (67%)	ILCT/MLCT	3.17	322.62	*0.1866*
299	H-6→LUMO (89%)	ILCT	3.19	320.90	*0.349*
	HOMO→L+2 (84%)	ILCT	3.48	293.87	*0.7349*
	H-4→L+3 (80%)	ILCT	3.60	284.48	*0.0144*
	H-7→L+1 (90%)	ILCT	3.85	265.79	*0.1787*
	H-1→L+2 (87%)	ILCT	3.93	260.82	*0.0321*
	H-9→LUMO (45%)<br>H-2→L+2 (44%)	ILCT<br>ILCT	4.04	253.16	*0.0456*
203	H-9→L+1 (35%)<br>H-6→L+2 (57%)	ILCT<br>ILCT/IL	4.40	232.68	*0.0532*
	HOMO→L+5 (57%)<br>H-5→L+2 (32%)	ILCT/IL<br>ILCT/IL	4.68	218.68	*0.0669*
	H-1→L+4 (62%)	ILCT/IL	5.04	203.29	*0.094*

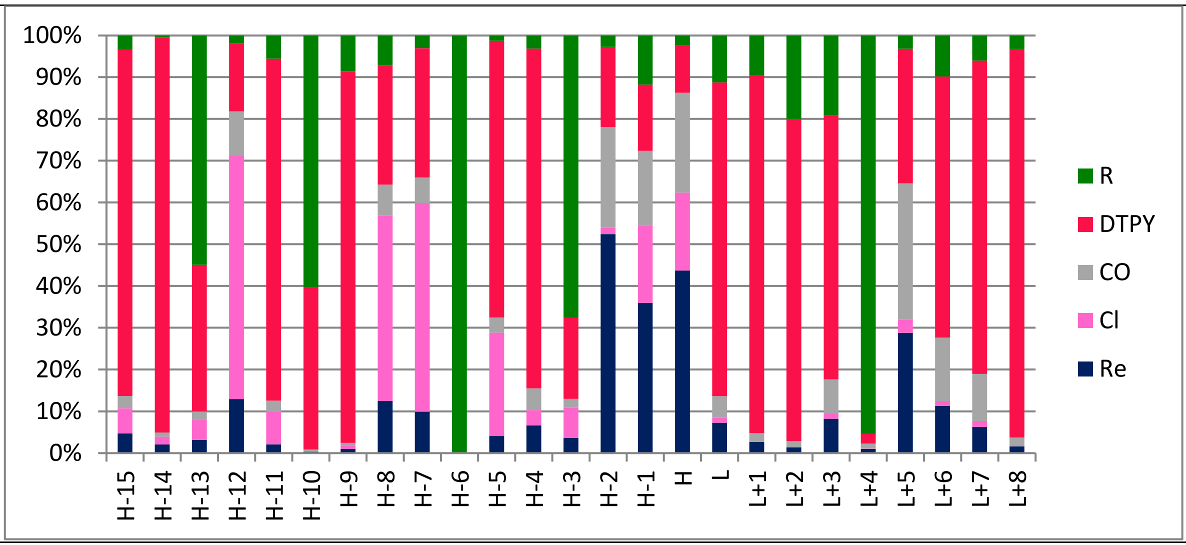

[ReCl(CO)$_3$(Ph-C≡C-dtpy)] (chloroform)

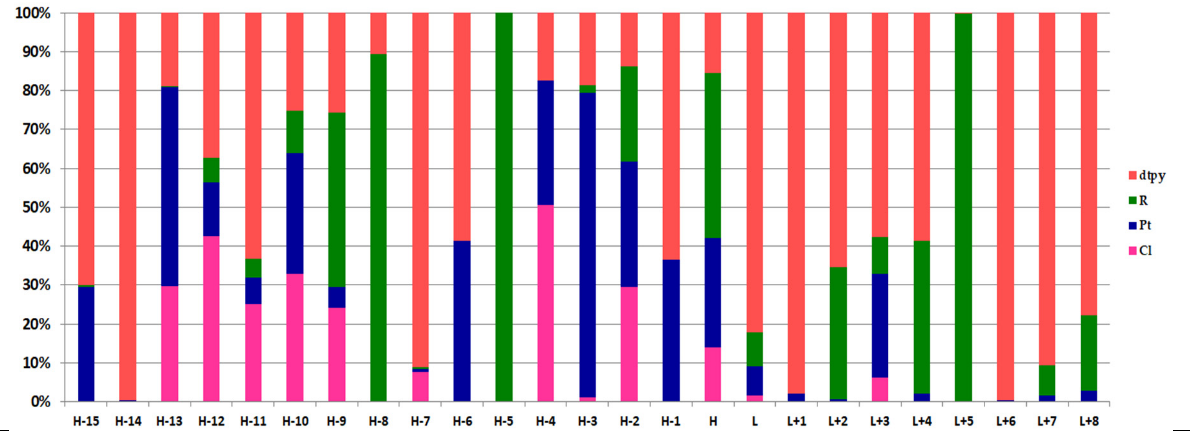

[Pt(Ph-C≡C-dtpy)Cl]CF$_3$SO$_3$ (acetonitrile)

Figure S3. Composition of frontier molecular orbitals of Re(I) and Pt(II) complexes

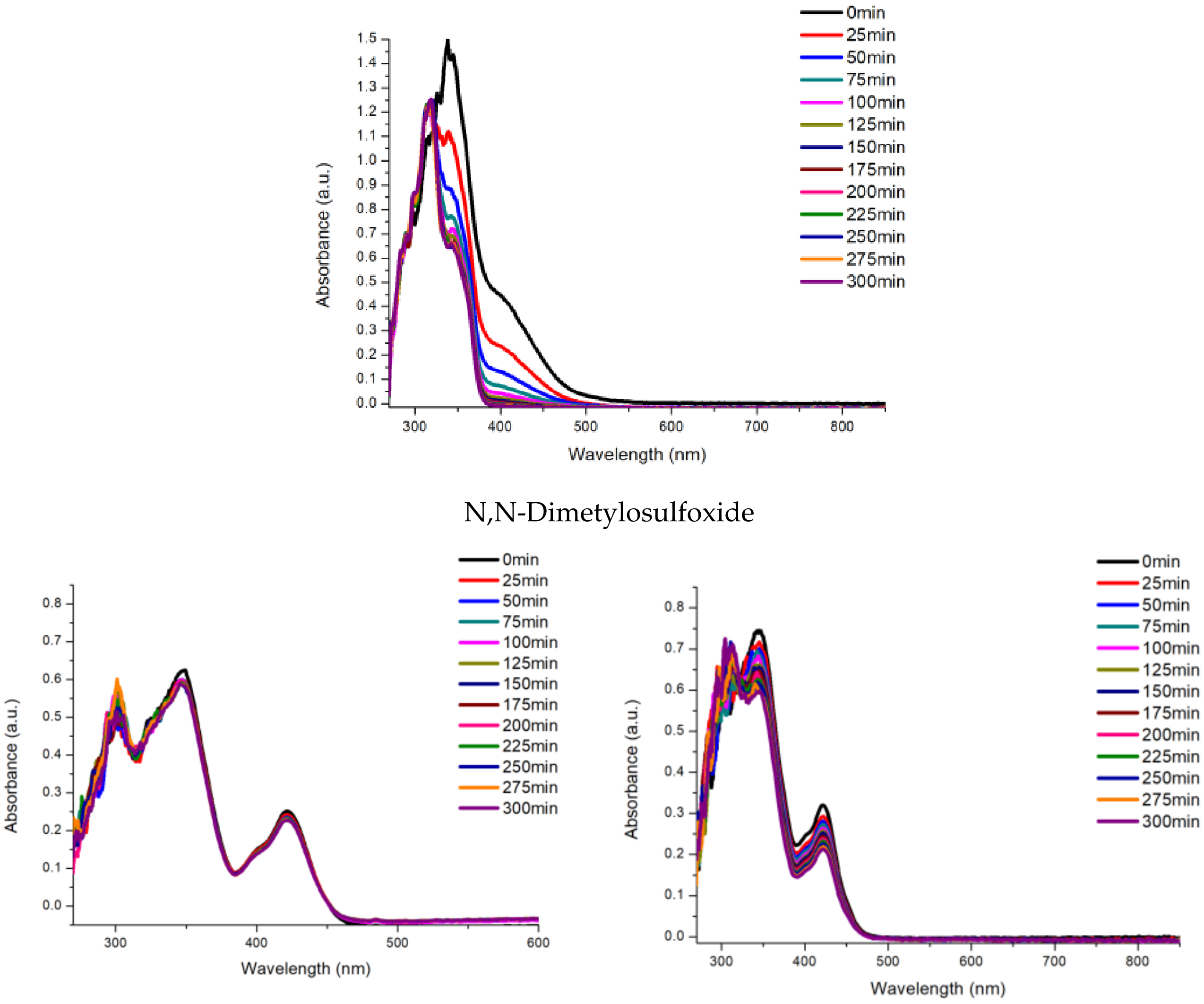

Re

N,N-Dimetylosulfoxide

Pt

Acetonitrile

N,N-Dimetylosulfoxide

Figure S4. UV-Vis absorption spectra of [ReCl(CO)$_3$(Ph-C≡C-dtpy)] in different solvents recorded once every 25 minutes for 5 h

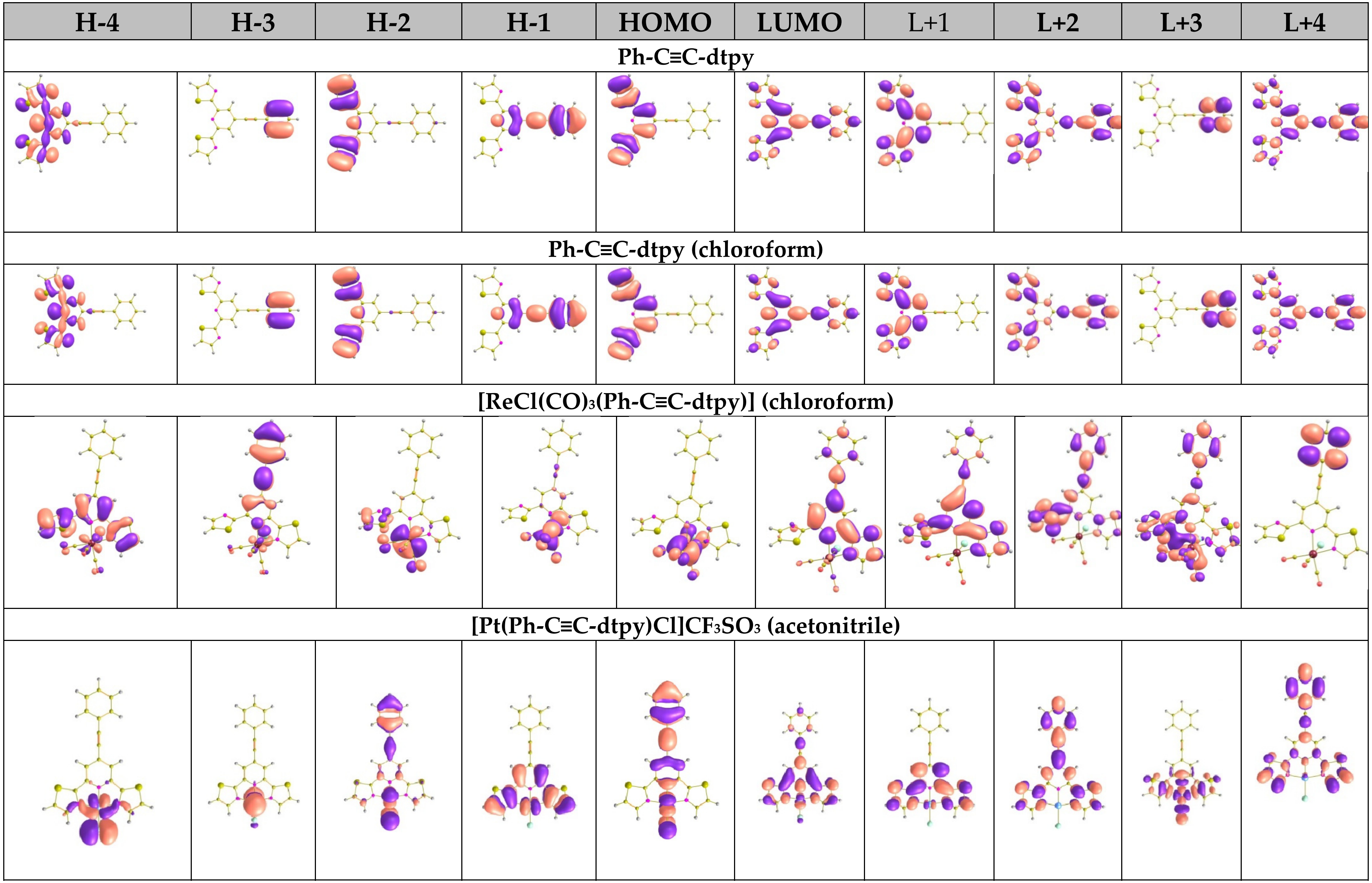

Figure S5. The contours of the frontier molecular orbitals

Figure S6. Luminescent properties of studied compounds in solid state, low temperature glass matrix (EtOH:MeOH, 4:1 v/v), acetonitrile and chloroform solutions. The lifetime decay curves were measured using EPL – 375 picosecond pulsed diode laser (Edinburgh Instruments), λ_{Ex} = 405nm

Medium	Emission and Excitation	Lifetime
	**Ph-C≡C-dtpy**	
CH_3CN	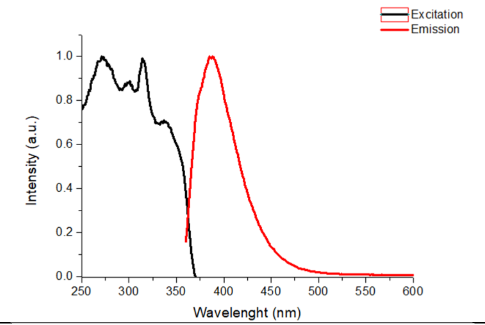	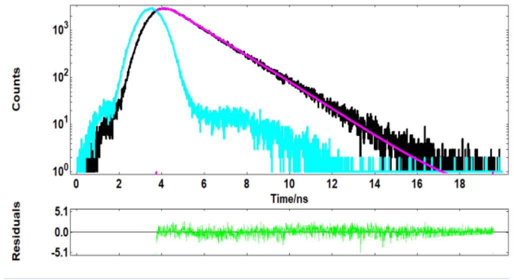
$CHCl_3$	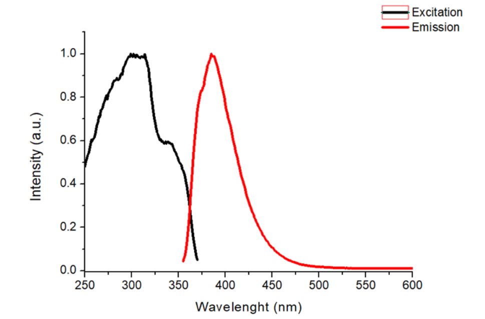	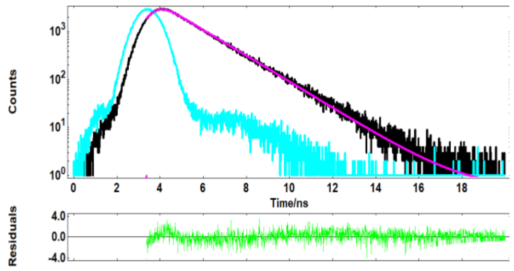
Solid	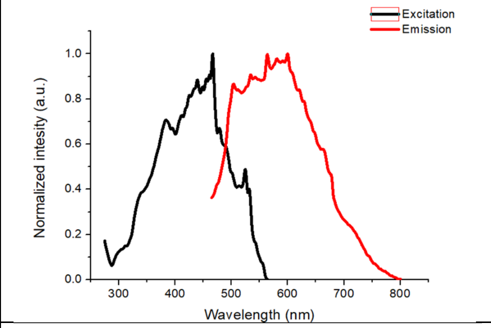	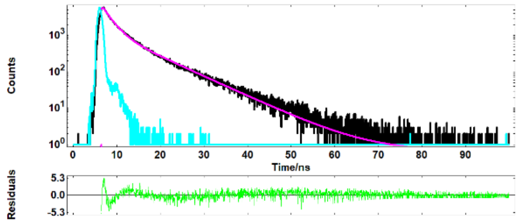
EtOH:MeOH (77K)	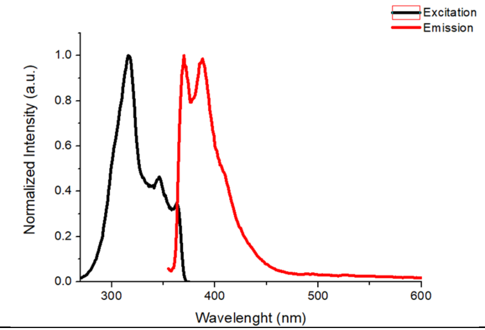	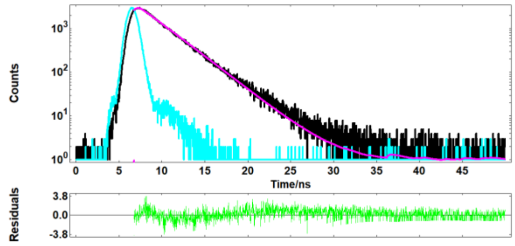

<table>
<tr><th colspan="3">$[ReCl(CO)_3(Ph\text{-}C{\equiv}C\text{-}dtpy)]$</th></tr>
<tr><td>CH_3CN</td><td colspan="2">-----</td></tr>
<tr><td>$CHCl_3$</td><td>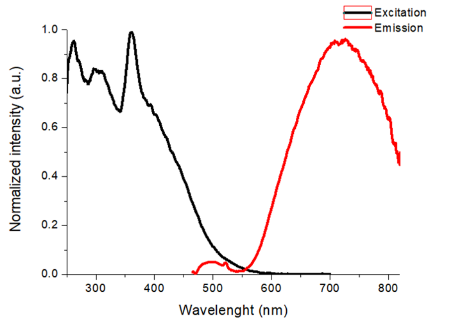
</td><td>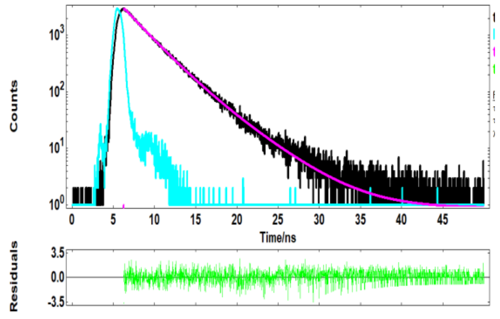
</td></tr>
<tr><td>Solid</td><td>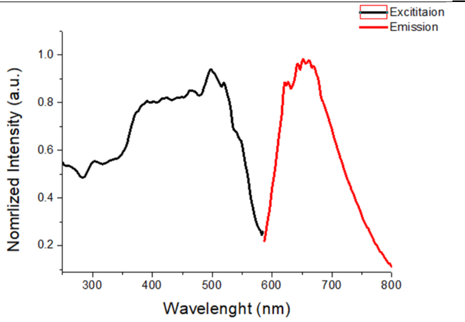
</td><td>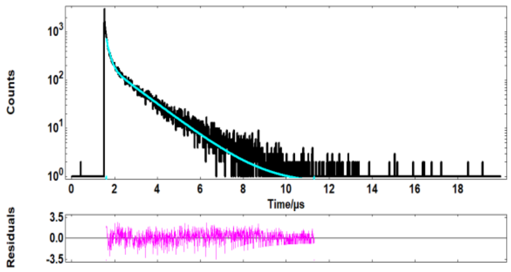
</td></tr>
<tr><td>EtOH:MeOH
(77K)</td><td>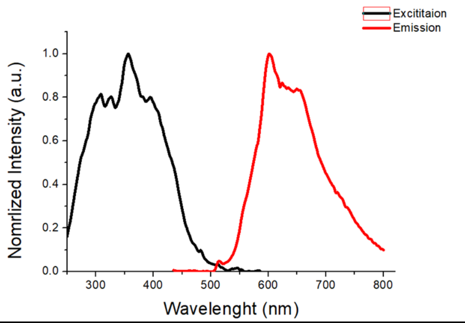
</td><td>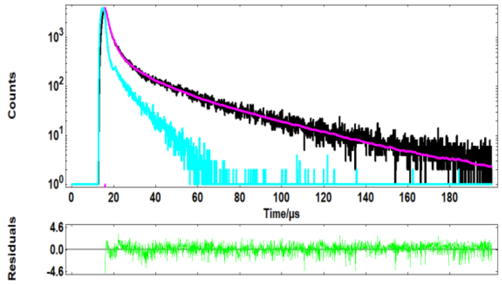
</td></tr>
</table>

[Pt(Ph-C≡C-dtpy)Cl]CF3SO3		
CH_3CN	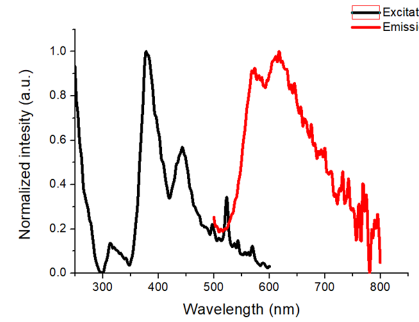	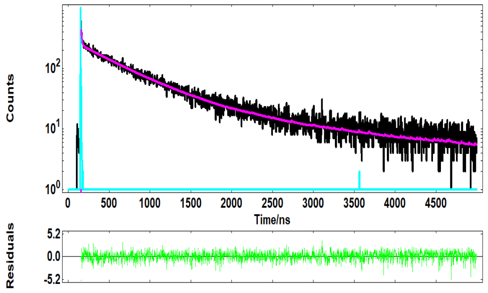
$CHCl_3$	-----	------
Solid	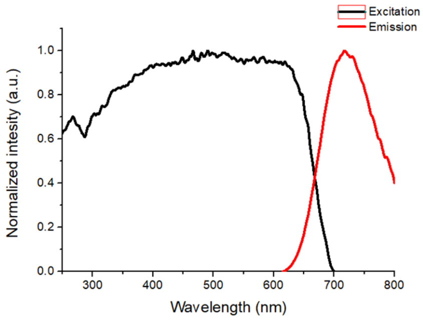	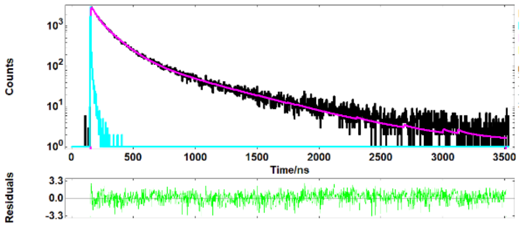
EtOH:MeOH (77K)	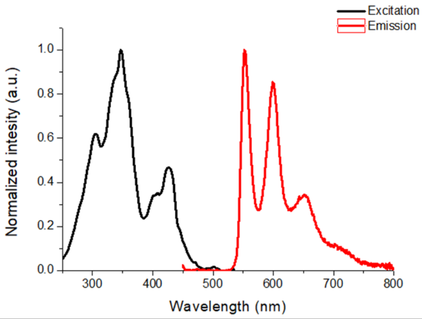	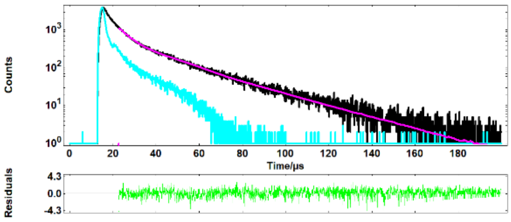

Table S9. TGA and DSC data

Compound	$T_{5\%}$ [°C]	$T_{10\%}$ [°C]	T_{max} [°C]	T_m [°C]
Ph-C≡C-dtpy	225	240	288	170
[ReCl(CO)3(Ph-C≡C-dtpy)]	277	325	601	–
[Pt(Ph-C≡C-dtpy)Cl]CF3SO3	344	397	474	–

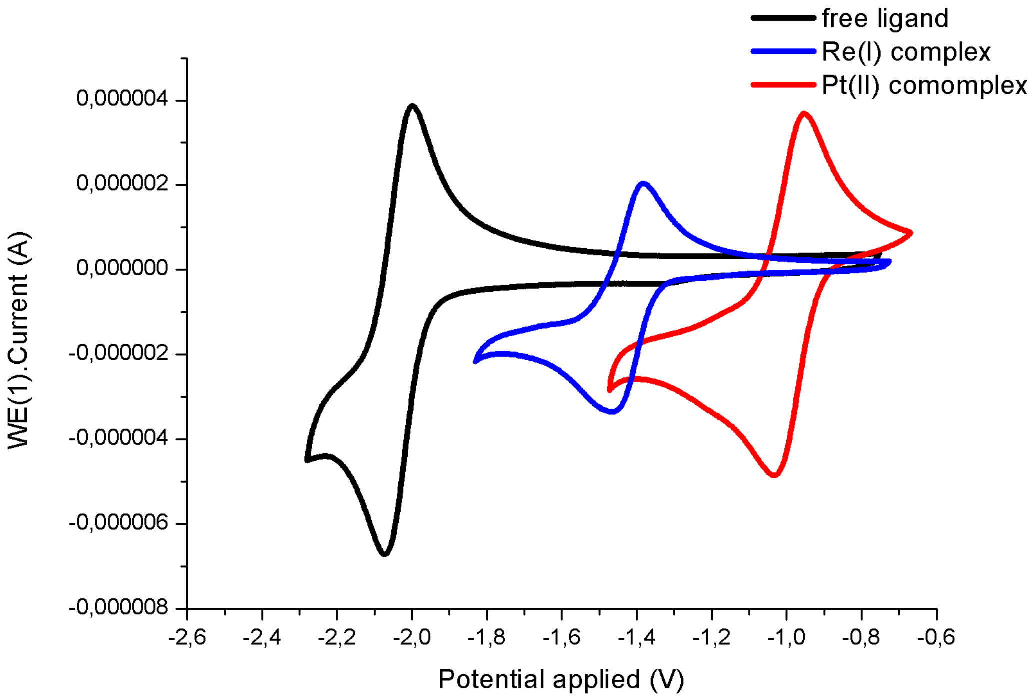

Figure S7. Cyclic voltammograms for free ligand and transition metal compounds

Table S10. Photophysical data for free dtpy-like ligands

Substituent	Solvent	Absorption data [nm]	Emission [nm]	Φ[%]	τ[ns]	Ref.
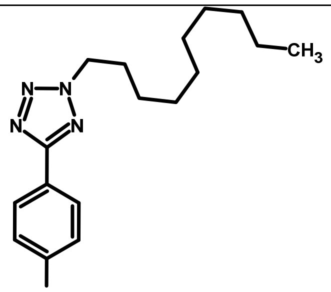	$CHCl_3$	239, 310, 332	385	15	1.66	[1]
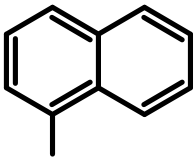	C_6H_{14}	242, 302, 325	359, 369, 371	41.09	2.24	[2]
	$CHCl_3$	303, 330	377	43.86	2.19 (96.10%), 0.27 (3.90%)	
	CH_3CN	251, 272, 324	386	32.95	1.96	
	CH_3OH	245, 302, 325	422	20.72	1.86	
	Solid	258, 297, 336, 363	386, 548	14.94	0.43 (85.51%), 1.20 (14.49%)	
		332, 354(sh), 393, 461, 483	536	brak	4.06 (89.51%), 1.06 (10.49%)	
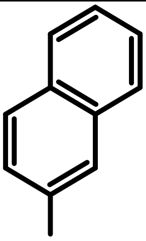	C_6H_{14}	267, 298, 333	361, 380, 400	28.86	2.07	[2]
	$CHCl_3$	260, 329	372, 385, 407	21.66	2.24 (96.45%), 0.58 (3.55%)	
	CH_3CN	275, 323	385	36.04	2.48	
	CH_3OH	246, 310, 333	392	35.78	3.06	
	Solid	255, 310, 336, 366	392	12.48	1.14 (65.48%), 0.49 (34.52%)	
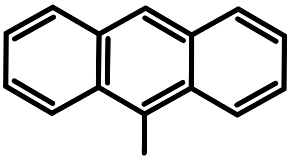	C_6H_{14}	245, 253, 301, 327, 344, 364, 383	407, 426, 457	41.78	3.31 (94.97%), 0.57 (5.03%)	[[2]
	$CHCl_3$	301, 332, 368, 387	440	21.36	2.34 (93.29%), 0.48 (6.71%)	
	CH_3CN	260, 300, 328, 344, 365, 385	463	13.41	2.32	
	CH_3OH	254, 297, 382	501	2.06	2.50 (85.87%), 0.02 (14.43%)	
	Solid	328, 350, 370, 395, 418, 448, 467, 480	540 (sh: 444, 498, 571)	13.04	3.72 (57.75%), 1.42 (39.26%), 0.02 (3.00%)	
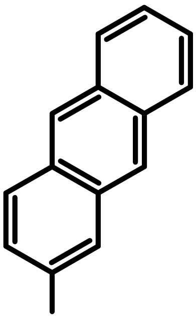	C_6H_{14}	244, 251, 300, 337, 355, 375	376, 401*, 427, 454, 487	10.85	4.91 (52.59%), 1.61 (47.41%)	[2]
	$CHCl_3$	252, 315, 345, 370, 394	452	43.30	4.22	
	CH_3CN	246, 263, 305, (sh: 343, 387)	474	56.43	6.92	
	CH_3OH	251, 298, 323, 338, 356	377, 399*, 423	19.35	4.04 (70.49%), 1.16 (29.51%)	
		265, 307, 347 (sh)	488	brak	brak	
	Solid	433	500, 532, 572	15.98	5.20 (93.51%), 1.79 (6.49%)	

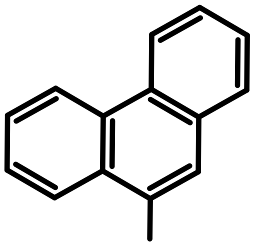	C_6H_{14}	252, 304, 324	361, 369*, 371, 388	14.17	5.00 (93.97%), 1.17 (6.03%)	[2]
	$CHCl_3$	265, 312, 330	383	31.24	2.54	
	CH_3CN	266, 325	403	23.38	1.94	
	CH_3OH	254, 304, 326	431	16.10	1.79	
	Solid	260, 302, 357	393, 544	27.83	0.26 (86.79%), 1.79 (13.21%)	
		335, 354, 383, 465	549		3.85 (67.49%), 1.41 (32.51%)	
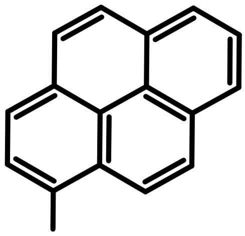	C_6H_{14}	242, 280, 344	392*, 409, 431 (sh)	50.99	3.24	[2]
	$CHCl_3$	281, 326, 351	439	62.69	2.41 (87.91%), 0.15 (12.09%)	
	CH_3CN	260, 309, 367, 379(sh)	458	64.52	2.90	
	CH_3OH	336, 365	483	21.45	3.20 (86.13%), 0.04 (13.87%)	
	Solid	379, 407	565	8.16	1.60 (52.01%), 5.53 (58.73%),	
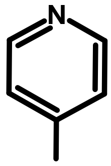	$CHCl_3$	336.8 ,305.7 293.8, 244.3	390	23.3	1.57	[3]
	CH_3CN	335.2, 302.6, 291.0, 238.9	392	22.8	1.74	
	Solid	-	417, 550	7.2	1.40 (76.06 %), 0.44 (23.94 %)	
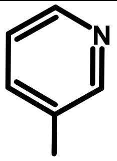	$CHCl_3$	333.4, 304.5, 291.1	381	23.3	1.30	[3]
	CH_3CN	332.1, 282.0	382	22.2	1.42	
	Solid	-	408, 541	11.6	1.04 (66.85 %), 0.10 (33.15 %)	
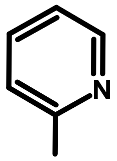	$CHCl_3$	346.1, 325.2, 290.6, 242.0, 239.7	385	22.9	1.34	[3]
	CH_3CN	338.74, 288.2, 231.4	386	22.5	1.49	
	Solid	-	423	9.7	1.80 (87.50 %), 0.16 (12.50 %)	
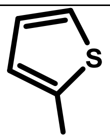	$CHCl_3$	355.0, 340.4, 303.9	381	22.4	1.91	[3]
	CH_3CN	352.9, 339.0, 299.4, 234.0	382	15.9	1.85	
	Solid	-	418, 552	7.1	1.15	
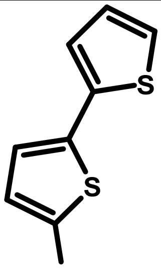	$CHCl_3$	365.9, 306.0	441	45.4	0.900.07 (24.22 %), 1.23 (75.78 %)	[3]
	CH_3CN	375.9, 314.3, 244.6, 205.3	459	43.0	0.74 (32.48 %), 2.63 (67.52 %)	
	Solid	-	531	3.4		
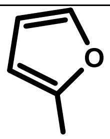	$CHCl_3$	354.7, 339.8 , 297.5	381	18.9	2.10	[3]
	CH_3CN	351.4 , 335.1 , 300.3, 229.7	380	26.7	2.50	
	Solid	-	424	14.8	1.77	

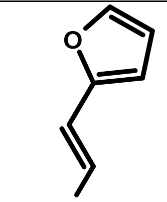	$CHCl_3$	343.2 , 301.7	409	5.3	0.18 (67.23 %), 2.22 (32.77 %)	[3]
	CH_3CN	346.6 , 316.3, 243.1, 204.0	445	2.7	0.02 (83.41 %), 3.07 (16.59 %)	
	Solid	-	473	5.2	1.71 (70.09 %), 0.31 (29.91 %)	
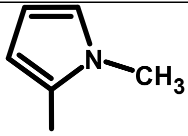	$CHCl_3$	343.0, 280	427	9.1	1.40	[3]
	CH_3CN	339. 6, 304.6, 226.2	493	4.7	2.46	
	Solid	-	433, 489	10.8	1.88 (71.63 %), 0.44 (28.37 %)	
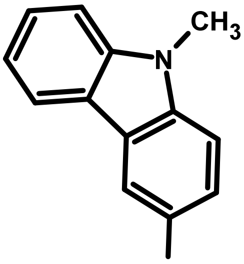	$CHCl_3$	377.4 , 350.3 , 334.8, 302.4 , 287.0, 269.4	424	48.2	1.87	[3]
	CH_3CN	369.8 , 330.1, 293.0, 237.6	470	35.0	3.54	
	Solid	-	428, 618	2.0	1.85 (69.66 %), 0.72 (30.34 %)	
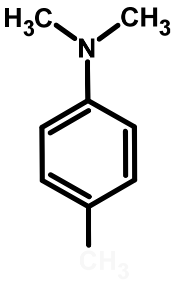	C_6H_{14}	345 , 321 , 299 , 268 sh, 227	-	-	-	[4]
	$CHCl_3$	359 , 325, 303, 278 sh, 235	468	24	2.14	
	CH_3CN	356 , 322, 300, 277 sh, 226	578	12	2.56	
	CH_3OH	363 , 321, 301, 280 sh, 227	-	-	-	
	Solid	-	591	8	0.95 (13.01%), 5.26 (86.99%)	
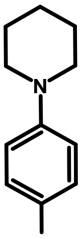	C_6H_{14}	337 , 323sh, 299, 271 sh,227	-	-	-	[4]
	$CHCl_3$	351, 325 sh, 284	488	35	2.52	
	CH_3CN	352 , 326 sh, 300, 275sh, 227	583	12	2.66	
	CH_3OH	350, 328 sh, 274	-	-	-	
	Solid	-	474	3	1.28 (65.68%), 4.55 (34.32%)	
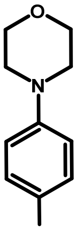	C_6H_{14}	329 , 305, 225	-	-	-	[4]
	$CHCl_3$	322, 299 sh, 289	461	34	1.98	
	CH_3CN	329 sh, 304 , 216	561	16	3.11	
	CH_3OH	331 sh, 305, 227	-	-	-	
	Solid	-	523	22	2.45	

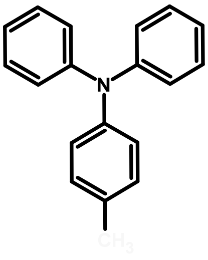	C_6H_{14}	369, 321, 293, 232	-	-	-	[4]
	$CHCl_3$	376 , 327sh, 297	488	81	3.84	
	CH_3CN	366, 326 sh, 294, 228	549	50	4.59	
	CH_3OH	374, 324sh, 295, 252 sh	-	-	-	
	Solid	-	565	20	4.01(31.64%),10.64 (68.36%)	
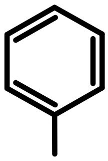	CH_3CN	332.5, 300.6, 285.6, 217.5	378	24.77	1.33	[5]
	$CHCl_3$	334.0, 302.9, 287.6	377	25.68	1.25	
	solid	-	393	4.47	0.90	
		-	557	23.42	5.31	
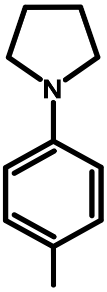	CH_3CN	371.9, 321.4, 301.8, 228.6, 194.4	580	7.09	2.33	[5]
	$CHCl_3$	373.4, 324.5, 304.0	475	23.40	2.42	
	solid	-	571	21.44	1.41 (20.49%), 3.47 (79.51%)	

Table S11. Photophysical data for tricarbonyl Re(I) compounds based on dtpy-like ligands

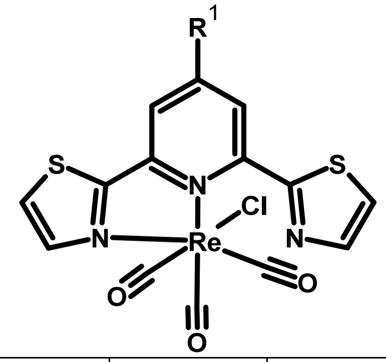

Substituent	Solvent	Absorption data[nm]	Emission [nm]	Φ[%]	τ[ns]	ref
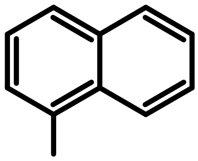	CHCl3	415, 326, 258	728	4.25	6.7 ns	[6]
			492 (I), 719 (II)		3.6 ns	
	77K	------	576	-	16800	
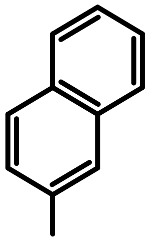	CHCl3	416, 324, 276, 267	731	3.72	6.7 ns	[6]
			490 (I), 729 (II)		5.9 ns (I)	
	77K	-----	533 sh, 573	-	10300 μs	
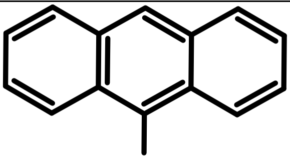	CHCl3	430 , 386, 365, 333	495	6,04	4.66	[6]
	77K	-----	499, 538, 599, 650 (sh) (I) 691, 766 (II)	-	4800 (I) 5045800(II)	
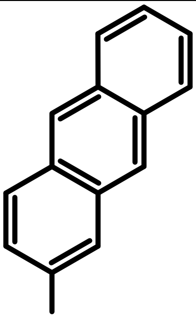	CHCl3	438, 373, 355, 326, 300	575 (I), 711 (II)		3160 (II)	[6]
			602		6.16	
	77K	----	700, 778	-	1996800	

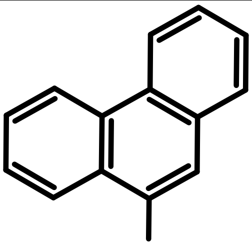	$CHCl_3$	413, 323, 297, 254	728	0.82	6.3	[6]
			502 (I), 727 (II)		2.2(I)	
	77K	---	586	-	4500	
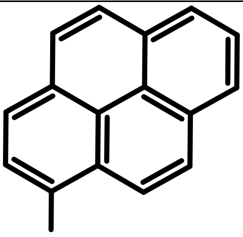	$CHCl_3$	426, 378, 331, 272, 264	540 (I) 723 (II)	6.11	3.43 (I) 1200 (II)	[6]
	77K	---	629, 692, 761	-	2626000	
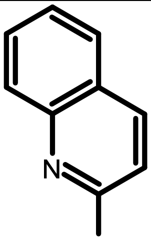	CH_3CN	397, 321 , 269	771	<0.01	2.4	[7]
	$CHCl_3$	418, 331 , 268	754	1.01	4.1	
	77K	-------	608	-------	1 518.9	
	solid	-----	648	4.16	3.1	
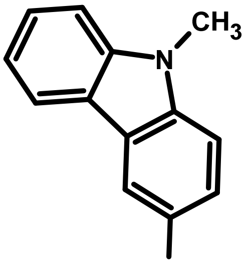	CH_3CN	411, 318, 290, 262, 245, 236, 229	711	0.79	5.7	[7]
	$CHCl_3$	418, 325, 293, 266, 246	712	1.23	8.5	
	77K	------	548, 574	----	6161.2	
	solid	------	635	3.50	85.1	
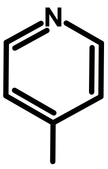	$CHCl_3$	432.3, 341.2 , 292.3	704	0.73	6.05 (14.70%), 37.55 (85.30%)	[3]
	CH_3CN	408.4, 335.8, 291.4, 266.2	703	0.98	13.50 (17.44%), 49.94 (82.56%)	
	77K	-	568	-	2698 (74.88%), 15 550 (25.12%)	
	solid	-	698	0.73	9.04 (47.39%), 28.70 (52.61%)	

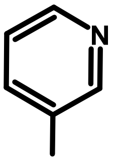	$CHCl_3$	428.6, 333.1, 278.7	717	0.51	7.29	
	CH_3CN	387.1, 326.2, 302.5, 220.4	726	0.49	5.02	
	77K	-	570	-	1763 (61.10%), 5279 (38.90%)	[3]
	solid	-	669	0.70	34.88 (72.93%), 274.06 (27.07%)	
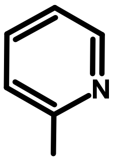	$CHCl_3$	435.2, 348.0, 306.9, 280.9	736	0.88	4.61	[3]
	CH_3CN	394.3, 331.4, 290.7, 266.2	756	0.34	3.16	
	77K	-	605		1116.66	
	solid	-	625	9.21	283.95	
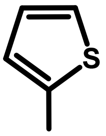	$CHCl_3$	433.1, 350.2, 321.4, 281.4	734	0.55	5.93	
	CH_3CN	382.5, 330.7, 298.3, 263.1	744	0.49	4.29	
	77K	-	535, 572	-	5200 (47.37%), 31 940 (52.63%)	[3]
	solid	-	640	3.15	293.44	
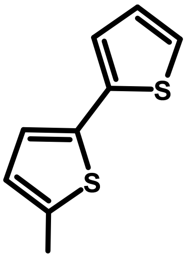	$CHCl_3$	431.3, 335.9, 296.0	691, 735	0.87	2.35 (3.22%), 198.82 (96.78%)	
	CH_3CN	419.5, 329.9, 264.0	682, 739	0.88	1.05 (40.58%), 155.07 (59.42%)	[3]
	77K	-	668, 738	2.96, 0.94	159.4 (2.95%), 122 950 (97.05%)	
	solid	-	694, 763	2.03	12 000 (22.02%), 58 210 (77.98%)	
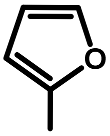	$CHCl_3$	418.9, 336.3, 305.9, 261.6	731	0.28	6.27	
	CH_3CN	395.8, 327.7, 247.0	720	0.80	4.08 (32.14%), 11.55 (67.86%)	
	77K	-	537, 579		11 840 (40.03%), 42 490 (59.97%)	[3]
	solid	-	630	4.52	120.00 (28.14%), 313.10 (71.86%)	

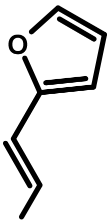	$CHCl_3$	418.4 sh, 380.6, 332.3, 262.2	682, 739	0.71	3.12 (7.43%), 126.86 (92.57%)	[3]
	CH_3CN	398.8, 366.9, 331.4, 260.3	671, 737	0.77	3.09 (22.34%), 139.23 (77.66%)	
	77K	-	656, 728	0.42	24 780 (39.58%), 69 700 (60.42%)	
	solid	-	671, 737	1.51	3672	
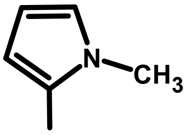	$CHCl_3$	411.9, 363.1, 324.4, 257.9	717	0.72	6.72	[3]
	CH_3CN	399.3, 356.0, 316.2, 250.9	716	0.50	2.39 (36.12%), 5.16 (63.88%)	
	77K	-	552, 588	0.31	13 090 (41.46%), 63 170 (58.54%)	
	solid	-	608	6.40	268.16 (38.20%), 591.88 (61.80%)	
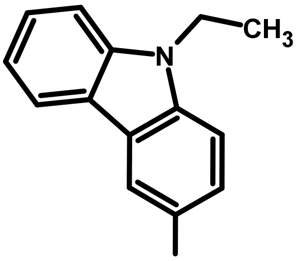	$CHCl_3$	417.9, 324.8, 293.0, 266.1, 246.2	706	0.88	8.26	[3
	CH_3CN	410.8, 318.4, 290.4, 262.3, 245.1, 236.1, 229.3	715	0.79	5.65	
	77K	-	541, 574	0.15, 0.78	4317 (46.04%), 28 140 (53.96%)	
	solid	-	695	3.72	85.54	
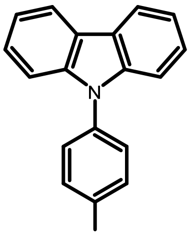	$CHCl_3$	415, 337, 289, 262, 246	734	0.77	5.78	[8]
	CH_3CN	394, 331, 287, 237	733	0.92	3.98	
	77K	-	578	-	2635 (56.68%), 9826 (43.32%)	
	solid	-	618	7.55	46.67 (38.05%); 300.57 (61.95%)	
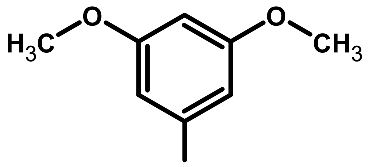	$CHCl_3$	394, 297	730	0.4	6.29	[9]
	CH_3CN	378, 331, 297	735	0.9	4.44	
	solid	-	630	3.8	362.08	

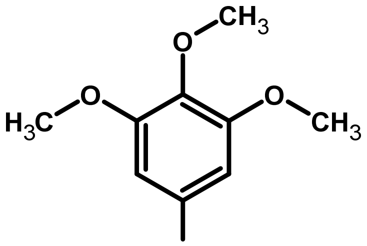	$CHCl_3$	401, 345, 257	702	0.3	6.28	[9]
	CH_3CN	398, 335, 30	703	0.4	4.79	
	solid	-	620	9.5	208.12	
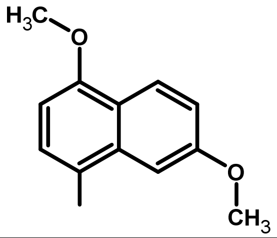	$CHCl_3$	419, 328, 248	717	1.3	6.99	[9]
	CH_3CN	397, 369, 297	722	1.0	4.79	
	solid	=	612	8.2	216.54	
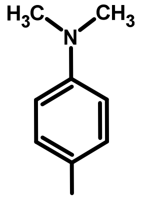	MeCN	436	732	0.7	7.1 ns (23.5%), 490 ns (76.5%)	[7]
	$CHCl_3$	443	697	0.3	13.0 ns	
	77K	-	596	-	66 μs (41.2%), 175 μs (58.8%)	
	solid	-	671	0.4	84.6 ns	
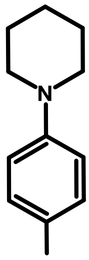	MeCN	432	750	3.2	9.0 ns (16.7%), 181.9 ns (83.3%)	[7]
	$CHCl_3$	442	701	0.6	12.6 ns	
	77kK	-	598	-	57.6 μs (37%), 153 μs (63%)	
	solid	-	660	0.1	1.4 ns (28.1%), 120 ns (12.4%), 851 ns (59.5%)	
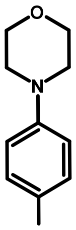	MeCN	415	727	0.2	9.3 ns (49.7%), 92.9 ns (50.3%)	[7]
	$CHCl_3$	424	716	0.9	8.9 ns	
	77K	-	578	-	3.3 μs (7%), 31.5 μs (35.1%), 95 μs (57.9%)	
	solid	-	682	1.6	326 ns (24.6%), 1.2 μs (75.4%)	
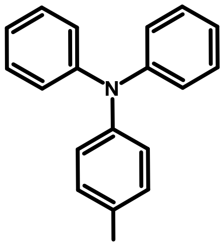	MeCN	436	734	0.6	41.6 ns	[7]
	$CHCl_3$	457	708	2.4	7.6 ns (96.3%), 451.4 ns (3.7%)	
	77K	-	579, 614sh	-	49 μs (51.8%), 154 μs (48.2%)	
	solid	-	609	2.5	28.1 ns (22.5%), 113 ns (77.5%)	

Table S12. Photophysical data for Pt(II) complexes based on dtpy-like ligands

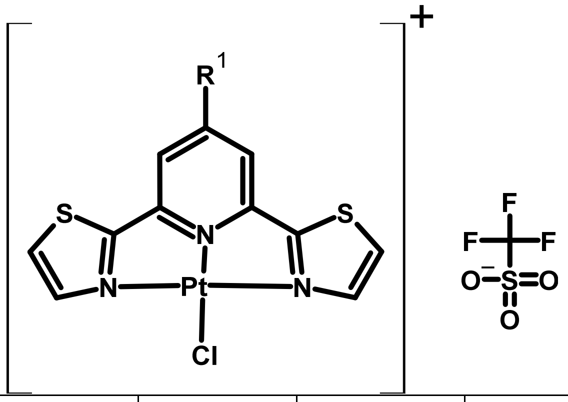

Podstawnik	Solvent	Uv-vis[nm]	Emissiom [nm]	Φ[%]	τ[ns]	ref
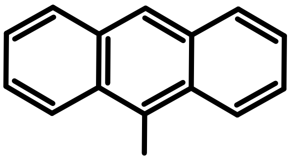	DMSO	443, 386, 365, 344, 335, 302, 262	512	6.30	13.45ns (84.57%), 2.92ns (11.72%), 0.03ns (3.71%)	[10]
	CH_2Cl_2	513, 417, 386, 366, 349, 308	755	-	10.53 (78.88%), 3.8 (21.12%	[11]
	77K	454, 385, 366, 347, 307	690, 768	-	1708.2μs (61.31%), 665.4 μs (38.69%)	
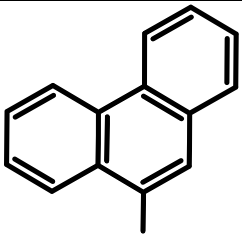	DMSO	417, 334, 299, 288, 262	472	3.87	6.62ns (6.15%), 2.30ns (45.58%), 0.62ns (48.27%)	[10]
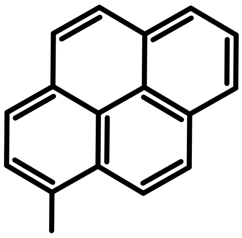	DMSO	444, 371(sh), 363(sh), 343, 329, 302, 276	570	12.16	3.39ns (100%)	[10]
	CH_2Cl_2	500, 417, 346, 306, 277	733	-	11.3 (68.44%), 4.2 (31.56%)	[11]
	77K	459, 430, 374, 322	480, 516, 566	-	12.32 ns (7.04%), 2.86 ns (80.81%), 0.54 ns (12.15%)	
		466, 388, 351, 318	660, 720, 777	-	286.6 μs (74.98%), 150.6 μs (25.02%)	

References:

1. Zych, D.; Slodek, A.; Małecki, J.G. 2,2′:6′,2′′-Terpyridine Derivative with Tetrazole Motif and Its Analogues with 2-Pyrazinyl or 2-Thiazolyl Substituents – Experimental and Theoretical Investigations. *Journal of Molecular Structure* **2020**, *1205*, 127669, doi:10.1016/j.molstruc.2019.127669.
2. Choroba, K.; Kula, S.; Maroń, A.; Machura, B.; Małecki, J.; Szłapa-Kula, A.; Siwy, M.; Grzelak, J.; Maćkowski, S.; Schab-Balcerzak, E. Aryl Substituted 2,6-Di(Thiazol-2-Yl)Pyridines –Excited-State Characterization and Potential for OLEDs. *Dyes and Pigments* **2019**, *169*, 89–104, doi:10.1016/j.dyepig.2019.05.015.
3. Klemens, T.; Czerwińska, K.; Szlapa-Kula, A.; Kula, S.; Świtlicka, A.; Kotowicz, S.; Siwy, M.; Bednarczyk, K.; Krompiec, S.; Smolarek, K.; et al. Synthesis, Spectroscopic, Electrochemical and Computational Studies of Rhenium(I) Tricarbonyl Complexes Based on Bidentate-Coordinated 2,6-Di(Thiazol-2-yl)Pyridine Derivatives. *Dalton Trans.* **2017**, *46*, 9605–9620, doi:10.1039/C7DT01948C.
4. Palion-Gazda, J.; Machura, B.; Klemens, T.; Szlapa-Kula, A.; Krompiec, S.; Siwy, M.; Janeczek, H.; Schab-Balcerzak, E.; Grzelak, J.; Maćkowski, S. Structure-Dependent and Environment-Responsive Optical Properties of the Trisheterocyclic Systems with Electron Donating Amino Groups. Dyes and Pigments 2019, 166, 283–300, doi:10.1016/j.dyepig.2019.03.035.
5. Klemens, T.; Świtlicka, A.; Szlapa-Kula, A.; Krompiec, S.; Lodowski, P.; Chrobok, A.; Godlewska, M.; Kotowicz, S.; Siwy, M.; Bednarczyk, K.; et al. Experimental and Computational Exploration of Photophysical and Electroluminescent Properties of Modified 2,2′:6′,2″-Terpyridine, 2,6-Di(Thiazol-2-Yl)Pyridine and 2,6-Di(Pyrazin-2-Yl)Pyridine Ligands and Their Re(I) Complexes. Applied Organometallic Chemistry 2018, 32, e4611, doi:10.1002/aoc.4611.
6. Maroń, A.M.; Palion-Gazda, J.; Szłapa-Kula, A.; Schab-Balcerzak, E.; Siwy, M.; Sulowska, K.; Maćkowski, S.; Machura, B. Controlling of Photophysical Behavior of Rhenium(I) Complexes with 2,6-Di(Thiazol-2-Yl)Pyridine-Based Ligands by Pendant π-Conjugated Aryl Groups. International Journal of Molecular Sciences 2022, 23, 11019, doi:10.3390/ijms231911019.
7. Klemens, T.; Świtlicka, A.; Szlapa-Kula, A.; Łapok, Ł.; Obłoza, M.; Siwy, M.; Szalkowski, M.; Maćkowski, S.; Libera, M.; Schab-Balcerzak, E.; et al. Tuning Optical Properties of Re(I) Carbonyl Complexes by Modifying Push–Pull Ligands Structure. Organometallics2019, 38, 4206–4223, doi:10.1021/acs.organomet.9b00517.
8. Choroba, K.; Maroń, A.; Świtlicka, A.; Szłapa-Kula, A.; Siwy, M.; Grzelak, J.; Maćkowski, S.; Pedzinski, T.; Schab-Balcerzak, E.; Machura, B. Carbazole Effect on Ground- and Excited-State Properties of Rhenium(I) Carbonyl Complexes with Extended Terpy-like Ligands. Dalton Trans. 2021, 50, 3943–3958, doi:10.1039/D0DT04340K.
9. Małecka, M.; Machura, B.; Świtlicka, A.; Kotowicz, S.; Szafraniec-Gorol, G.; Siwy, M.; Szalkowski, M.; Maćkowski, S.; Schab-Balcerzak, E. Towards Better Understanding of Photophysical Properties of Rhenium(I) Tricarbonyl Complexes with Terpy-like Ligands. Spectrochimica Acta Part A: Molecular and Biomolecular Spectroscopy 2020, 231, 118124, doi:10.1016/j.saa.2020.118124.
10. Choroba, K.; Machura, B.; Raposo, L.R.; Małecki, J.G.; Kula, S.; Pająk, M.; Erfurt, K.; Maroń, A.M.; Fernandes, A.R. Platinum(II) Complexes Showing High Cytotoxicity toward A2780 Ovarian Carcinoma Cells. Dalton Trans. 2019, 48, 13081–13093, doi:10.1039/C9DT02894C.
11. Maroń, A.M.; Choroba, K.; Pedzinski, T.; Machura, B. Towards Better Understanding of the Photophysics of Platinum(II) Coordination Compounds with Anthracene- and Pyrene-

Substituted 2,6-Bis(Thiazol-2-Yl)Pyridines. Dalton Trans. 2020, 49, 13440–13448, doi:10.1039/D0DT02650F.
